# A systematic review of shared decision making training programs for general practitioners

**DOI:** 10.1186/s12909-024-05557-1

**Published:** 2024-05-29

**Authors:** Jasmien Jaeken, Cathoo Billiouw, Lien Mertens, Pieter Van Bostraeten, Geertruida Bekkering, Mieke Vermandere, Bert Aertgeerts, Laura van Mileghem, Nicolas Delvaux

**Affiliations:** Department of PH&PC, Academic Center for General Practice, KU Leuven, Kapucijnenvoer 7 block h, box 7001, Leuven, 3000 Belgium

**Keywords:** Shared decision making, Decision making, Educational intervention, General practitioner

## Abstract

**Background:**

Shared decision making (SDM) has been presented as the preferred approach for decisions where there is more than one acceptable option and has been identified a priority feature of high-quality patient-centered care. Considering the foundation of trust between general practitioners (GPs) and patients and the variety of diseases in primary care, the primary care context can be viewed as roots of SDM. GPs are requesting training programs to improve their SDM skills leading to a more patient-centered care approach. Because of the high number of training programs available, it is important to overview these training interventions specifically for primary care and to explore how these training programs are evaluated.

**Methods:**

This review was reported in accordance with the PRISMA guideline. Eight different databases were used in December 2022 and updated in September 2023. Risk of bias was assessed using ICROMS. Training effectiveness was analyzed using the Kirkpatrick evaluation model and categorized according to training format (online, live or blended learning).

**Results:**

We identified 29 different SDM training programs for GPs. SDM training has a moderate impact on patient (SMD 0.53 95% CI 0.15–0.90) and observer reported SDM skills (SMD 0.59 95%CI 0.21–0.97). For blended training programs, we found a high impact for quality of life (SMD 1.20 95% CI -0.38-2.78) and patient reported SDM skills (SMD 2.89 95%CI -0.55-6.32).

**Conclusion:**

SDM training improves patient and observer reported SDM skills in GPs. Blended learning as learning format for SDM appears to show better effects on learning outcomes than online or live learning formats. This suggests that teaching facilities designing SDM training may want to prioritize blended learning formats. More homogeneity in SDM measurement scales and evaluation approaches and direct comparisons of different types of educational formats are needed to develop the most appropriate and effective SDM training format.

**Trial registration:**

PROSPERO: A systematic review of shared-decision making training programs in a primary care setting. PROSPERO 2023 CRD42023393385 Available from: https://www.crd.york.ac.uk/prospero/display_record.php?ID=CRD42023393385.

**Supplementary Information:**

The online version contains supplementary material available at 10.1186/s12909-024-05557-1.

## Introduction

Almost all patients want to be actively involved in decisions related to their health care during a clinical encounter with their physician [1, 2]. They want physicians to explain the benefits and risks of a health care decision specific to their individual problem and to discuss their preferences and values with them. In contrast to this desired role, only 39% of patients feel actively involved whereas 37% feel less involved than they would like to be [2, 3]. This illustrates that physicians are not addressing the needs of patients with regard to their wish to play an active role in decision-making regarding their health and health-related choices.

Shared decision making (SDM) has been presented as the preferred approach for decisions where there is more than one acceptable option. It is a process in which both patient and physician communicate the risks and benefits of a health care decision and where a decision is made based on clinical guidelines taking into account the patients’ values, concerns and preferences [4, 5]. The key elements of SDM include the following: definition/explanation of the problem, presentation of options, discussion of pros/cons, exploration of patient values/preferences, discussion of patient ability/self-efficacy, presentation of doctor knowledge/ recommendations, clarification of understanding, making or explicitly deferring decisions, and arranging follow-up [6]. SDM has been shown to improve health outcomes such as quality of life and patient satisfaction. It also strengthens the patient-physician relationship and patients feel more understood or appreciated [7, 8].

Although physicians are aware of patient’s preferences for SDM, they often fail to elicit patients’ values and lack the skills to involve patients during a consultation [9]. Overall, patient involvement in discussing treatment options is limited by their pros and cons and insufficient attention is given to involving patients in the decision-making process such as asking about their preferences and concerns [10]. Many physicians think that they already adequately involve patients however, they do not see how SDM differs from their usual consultation style, indicating that physicians do not always have a clear understanding of what SDM implies [11]. This mismatch can lead to unmet expectations of treatment outcomes and decreased patient satisfaction [12]. Furthermore, approximately half of the physicians report insufficient communicative competence in applying SDM in cancer screening programs and only 8% feel qualified to implement these skills [13]. In addition to this clinician uncertainty in self-competence, other physician-reported barriers to SDM use include lack of familiarity with SDM and insufficient level of SDM training [9]. To answer these barriers, training physicians in SDM has been proposed as part of the puzzle toward better implementation in daily practice. SDM training has a positive effect on SDM skills in daily practice as does improved communication skills, an increased positive attitude toward patient involvement and patient participation [14, 15]. Furthermore, physicians trained in SDM may continue to integrate patient-involving behaviors over time [16, 17]. Physicians also report increased confidence and comfort in SDM resulting from a training intervention [18–20]. However, there is no consensus about the core competencies an SDM training program should contain, indicating another important gap in current SDM research [21].

General practitioners (GPs) are characterized by longstanding and familiar relationships with patients and their families leading to a foundation of trust and advocacy for a holistic approach to health problems. They play a central role in the somatic and psychosocial wellbeing of patients. Therefore, in a primary care setting SDM has important potential in delivering patient-centered care given the spectrum of health conditions encountered and the diversity of medical decisions made daily. However, most GPs do not feel confident enough to engage in SDM and request specific communication training to increase their SDM skills [22, 23]. To date, a wide range of SDM training programs have been developed to overcome the existing barriers and facilitate the use of SDM in clinical practice. The aim of this review is to provide an overview of current SDM training programs for GPs and to evaluate their effectiveness to inform future developers of SDM training programs.

To achieve this aim, the following research questions were posed:

1. How is SDM taught within training programs for general practitioners?
2. What is the effectiveness of training general practitioners in SDM based on the Kirkpatrick evaluation model?
3. Is there a difference in the effectiveness of training based on training format?

**Table:** 

Why is it important to do this review?	
Patient involvement in decision making during healthcare consultations is a priority feature of high-quality patient-centered care. Considering the foundation of trust between GPs and patients and the variety of (chronic) diseases in primary care, the primary care context can be viewed as roots of SDM application. General practitioners request communication training programs to improve their SDM skills for a more patient-centered care approach. Because of the high number of training programs available, it is important to summarize these training interventions specifically for primary care and to evaluate their effectiveness.	

## Methods

### Study design

We conducted a systematic review adhering to the reporting guidelines of the preferred reporting items for systematic reviews and meta-analyses (PRISMA) statement [24].

### Literature search

We performed an electronic search on 9th December 2022 and again in September 2023, using following databases: Medline (via Pubmed), EMBASE, CINAHL, Web of Science, SCOPUS, Cochrane Central Register of Controlled Trials (CENTRAL) and ERIC (Additional file 1). We manually reviewed the reference lists of all included studies and relevant systematic reviews. The following ‘grey literature’ sources were used: ANZCTR (Australian New Zealand clinical trials registry), ClinicalTrials.gov, International Clinical Trials Registry Platform (ICTRP), AMEE (Association Medical Education Europe) and NVMO (Dutch association in medical education). The keywords used were: “Primary care physicians”; “General practitioners”; “patient-centered care”; “shared decision making”; “Training” and “educational interventions”. We also hand-searched the proceedings of the International Conference on Shared Decision Making (from 2003 to 2022) and the proceedings of the annual North American Meetings of the Society for Medical Decision Making and we consulted experts in this research field using an SDM Facebook group. The first author (JJ) received weekly emails of the search query of the different databases to update the reference list. There were no restrictions on geographical region, time frame or language. An expert librarian was involved to validate the search strategy. Endnote was used to keep track of the selected literature and to remove duplicates. We uploaded the de-duplicated search results to Covidence©.

### Inclusion and exclusion criteria

#### Population

We included studies involving SDM training interventions developed for general practitioners. Studies describing training interventions for medical undergraduates or patients, nurse practitioners or physician assistants, and interventions specific and solely for secondary care physicians were excluded from this review. Studies describing a broad population like ‘Physicians’ or ‘Health care providers’ were included. Studies involving both primary and secondary care physicians were included if the training concerned the overall aspect of training SDM skills.

### Types of intervention

To be eligible for this review, the SDM training program needed to meet the following criteria: (1) to have the aim to actively involve patients in the decision-making process (2), to offer a training intervention in SDM for general practitioners (3), a clear description of the learning module used and (4) an evaluation of the training program. We also included studies that did not explicitly define SDM but incorporated a balanced discussion of the pros and cons of a health care decision with the patient’s values to facilitate an informed decision. Studies describing training interventions for basic overall communication skills or training modules that did not have the primary aim to train SDM communication skills were excluded from this review. We excluded articles where only health related outcomes (e.g. blood pressure, lipid levels…) are measured (no outcomes related to training intervention or measuring SDM skills acquired after training).

#### Study selection

The database search was conducted by four members of the research team (ND, GB, LvM and JJ). During the first screening round, titles and abstracts were screened for inclusion according to the eligibility criteria by the four members. The screening process was first piloted by discussing the in- and exclusion criteria applied on the first 50 articles. When the reviewers disagreed about including an abstract, the full text was considered. Inclusion of studies at both levels (abstract and full text) were independently assessed and discrepancies were resolved through consensus by two review participants (JJ and LvM). If consensus could not be reached, a third researcher was involved (ND). Reasons for non-eligibility were documented by the reviewers. Post hoc we decided to only use randomized controlled trials (RCTs) for further analysis in this review due to the high yield in articles.

#### Data extraction and quality assessment

One researcher (JJ) extracted the following data from the selected articles: title, authors, year of publication, country of study, type of study, study methodology, participant characteristics, SDM program name, date of program development, format, duration of training, length of follow-up, evaluation measures of training and SDM skills. Data extraction sheets were first pilot-tested and adjusted if necessary. Missing data were recorded and, where applicable, the authors were contacted for clarification.

### Risk of bias assessment

The ICROMS (Integrated Quality Criteria for Review of Multiple Study designs) tool was used to assess the quality of the included studies [25]. We used ICROMS because it allowed us to assess the quality of diverse study designs, including randomized studies, controlled before-and-after studies and interrupted time series, and it incorporates criteria for non-controlled before-and-after studies, cohort studies and qualitative studies. The risk of bias was assessed in duplicate and independently (JJ and CB). Any disagreements were resolved by consensus. If consensus was not achieved, a third reviewer was consulted (ND). Post hoc we decided to only use RCTs for further analysis in this review but we did not change the Risk of Bias Tool.

### Data analysis

We categorized the studies according to training format: (1) online learning (2), live learning and (3) blended learning. We performed a meta-analysis of the included RCTs. We analyzed all data with a random-effects model because of the heterogeneous nature of the interventions. When the study reported repeated measurements for an outcome for the same participants, only the measure closest in time to the training was kept in the meta-analysis. For categorical data, we calculated the risk difference. We calculated standardized mean difference (SMD) for continuous measures, and we considered the Cohen’s criteria to assess if the there was a small (Cohen’s d < 0.2), medium (Cohen’s d 0.2–0.5) or large (Cohen’s d > 0.8) effect size. Data was analyzed using Revman [26].

### Types of outcome measures

We included all reported effect measures to describe effect size in included studies of quantitative outcomes (e.g., mean difference or risk difference with appropriate confidence intervals). Reported outcomes were summarized and categorized into patient-reported, observer-reported, or physician-reported SDM. We also included satisfaction, decisional conflict, decisional regret and quality of life. Furthermore, we connected these outcomes with Kirkpatrick’s evaluation framework for healthcare provider trainings in SDM [27]. Kirkpatrick’s four-level training evaluation model is the most feasible model for training evaluation and can provide a better understanding of the impact and value of the training program:

1. health care providers’ reactions (satisfaction with training, objective training acceptability and feasibility, quality rating);
2. health care providers’ learning (self-reported competence with SDM and knowledge);
3. health care providers’ behavior (provider- or observer-reported patient interaction e.g. SDM-Q9, OPTION scale); and.
4. health care system effects or patient health outcomes.

## Results

### Study selection

**Fig. 1:**
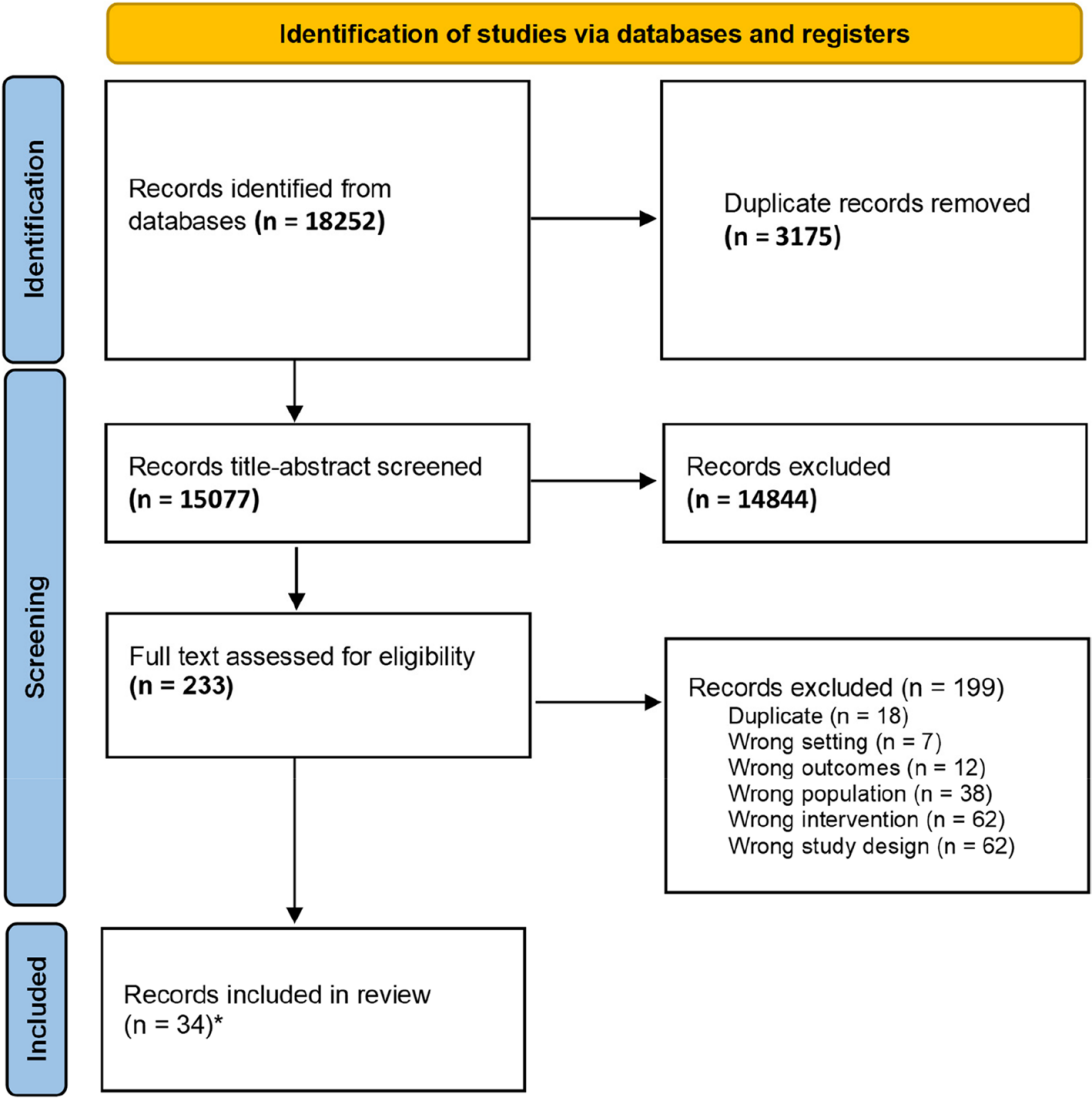
PRISMA flowchart. *34 records representing 29 studies

The literature search initially yielded 18,252 records. After removing duplicates, 15,077 unique records were identified. After screening the abstracts, 14,844 records were excluded because they did not meet the eligibility criteria. From the reviewed abstracts, 233 records were reviewed in full text. Of these, 34 final records were identified (Fig. 1).

### Characteristics of included records

The 34 records identified represent 29 studies. Of these 29 studies, 17 reported a live learning format [28–44], 10 studies [45–54] an online learning format and two reported a blended learning format [55, 56]. The most represented countries were the USA (*n* = 13 studies) and Germany (*n* = 5 studies). Seventeen studies referred to a theoretical framework on which their training program was based. The clinical conditions that were used most often for training were cardiovascular diseases (*n* = 9), cancer screening (*n* = 6) and diabetes (*n* = 5). The characteristics of the studies with training content can be found in Table 1 at the end of the review.

**Table 1:** Study characteristics of all included randomized controlled trials

Author (year) Country	Training format	Design	Training intervention Clinical setting	Outcomes used for analysis	Theoretical framework
Adarkwah (2016) [30] Germany	Live learning	C: General practitioners (GPs) took part in **meetings** to be trained in study procedures and their use. Patients were randomized to consultation with the TTE illustration.	GPs took part in **meetings** to be trained in the use of both ways of showing risk in arribaTM, the emoticons and their developed TTE. Each GP received **advice** on how to communicate risk according to the respective risk format. Finally, GPs received a **booklet** summarizing the content of the training for further reading and individual preparation. Clinical context: prevention of cardiovascular diseases	PEF-FB9 – German questionnaire for SDM Decisional conflict	Not specified
		I: GPs took part in **meetings** to be trained in study procedures and the use. Patients were randomized to consultation with the emoticons The main objective was to evaluate the non-inferiority of the new TTE illustration compared to the emoticons regarding their impact on SDM.			
Bakhit (2018) [45] Australia	Online	C: No intervention (usual care)	A USB-drive containing a 15-min **video-based SDM training package** that explained what SDM is, its use in acute respiratory infection consultations, and a consultation demonstrating use of one of the decision aids. Clinical setting: Acute respiratory infections; acute otitis media, acute sore throat and acute bronchitis	Decisional conflict OPTION-12 scale CollaboRATE-5 scale	Not specified
		I: The intervention consisted of patient decision aids (DA) for 3 health problems (acute otitis media, sore throat, acute bronchitis) and 15-minute video-delivered training for GPs			
Branda (2013) [46] USA	Online	C: No intervention (usual care)	Clinicians received training on how to use the conversation aid during a 10 min group session (including rationale, demonstration of use, role playing), Brief video clips and storyboards that demonstrate the basic use of decision aids. A study team member will remain available to do one-on-one demonstrations after the initial group demonstration if needed. Clinical setting: Diabetes and statins (CV prevention)	OPTION scale	RE-AIM framework: This framework has been developed specifically to address how an intervention, in this case use of decision aids, is implemented in a real-world setting.
		I: The intervention will consist of the use of a DA (Statin Choice and Aspirin Choice, **or** Diabetes medication Choice) by patients and their clinician during the clinical encounter. Training = clinicians how to use the DA.			
Cals (2007) [31] The Netherlands	Live learning	C: no intervention (usual care)	Consultation with simulated patient during routine consultation session before a training seminar (= T1) A communication expert moderated the seminars: reflection and feedback transcripts of T1. Outlining of the elicit-provide-elicit framework: the GP elicits what the patient knows about his condition and what the patient’s main worries and expectations are. Then the GP elicits the patients’ interpretation about what has been said and done. A video demonstration of the elicit-provide-elicit approach was presented and contrasted with a video example of a more ’common practice’ consultation. The GPs then had the opportunity to practice these skills with SPs. Clinical setting: respiratory tract infections	Patient satisfaction with consultation	Framework developed by Rollnick et al. The skills training was based on a patient-centred strategy to achieve shared decision about investigation and treatment of acute infections using the ‘elicit-provide-elicit’ framework.
		I: Enhanced communication skills training for GPs			
Cooper (2011) [47] USA	Online	**C**: Patient and physician minimal intervention	**Simulated visit with a patient was video recorded** **An interactive CD-ROM** using a videotape of each study **physician’s interview with a simulated patient** ◊ The software allows the physician to navigate the interview in an efficient manner and quickly review examples of specific skills. The physician receive **individualized feedback** **A workbook** that accompanies the CD-ROM provides an orientation to the RIAS analysis approach; and includes case-based exercises (include a review of their skills in five areas for improving patient adherence (eliciting the full spectrum of patient concerns; probing patients regarding their knowledge and beliefs about hypertension; monitoring patient adherence; assessing obstacles and resources, and eliciting a commitment to the therapeutic plan). Clinical setting: cardiovascular disease	Patient Ratings of Their Clinicians Participatory Decision-Making Skills Patient perceived involvement in care Patient-physician communication behaviour scale	Communication skills relevant to increasing patient engagement, activation, and empowerment organized within the context of the four functions of the medical interview (data-gathering, patient education and counseling, rapport-building, and facilitation and patient activation).
		**I**: Physician communication skills training by CD rom. Intervention was physician and patient directed randomized in 4 groups: (A) Physician + patient intervention. (B) Physician intervention only (C)Patient intervention only (D) Physician and patient minimal intervention = control			
Cooper (2013) [48] USA	Online	C: no intervention (usual care)	2 h lecture about clinical management of depression. Case-based interactive multimedia CD-ROM communication skills training program. The CD-ROM contained clinician’s interview with a simulated patient at baseline, analyzed using RIAS. Individualized feedback regarding communication was provided along with a companion workbook to introduce them to the software and guide through the cases. Clinical setting: depression	QOL	Not specified
		I: patient-centered collaborative care Patients in both control and intervention group were asked to try two educational materials.			
Den ouden (2022) [33] The Netherlands	Live learning	C: no intervention (usual care)	The GP’s are trained in SDM during a 2 h training session: The study protocol was discussed and SDM principles were reviewed to foster a common understanding of SDM processes. The OPTIMAL decision support tool was discussed By use of role-plays, the SDM process will be practiced by GPs Clinical setting: diabetes	Control preference scale OPTION scale	Framework of Montori et al.
		I: GP’s are trained in SDM during a 2 h training session. Patients were given a decision aid			
Elwyn (2004) [32, 68, 69] UK	Live learning	C: physicians first completed a Risk communication aid, then an SDM workshop	**SDM workshops**: Background literature on SDM and participants asked to debate its relevance to clinical practice. The skills (competences) of SDM were described and demonstrated using simulated consultations. Consult with the simulated patients using pre-prepared scenarios involving the study conditions. Participants were asked to consider the competences in more depth. All participants had conducted and received feedback from at least one consultation with a simulated patient. Participants also followed a Risk communication workshop. Clinical setting: cardiovascular disease, menopause, menorrhagia and prostatism	OPTION scale COMRADE scale Quality of life	The extended model of interpersonal interaction outlined by Hargie
		**I**: physicians first completed an SDM workshop, then a Risk communication aid			
Feng (2013) [49] USA	Online	**C**: Physicians received a brochure on PSA screening	**Web-based**: interactive roulette wheels Illustrative video vignettes Prostate cancer screening basic information Methods of SDM Clinical setting: PSA screening	Self-reported rate of SDM	Not specified
		**I**: Arm A: Web based tool for physicians Arm B: patient intervention			
Haskard (2009) [34] USA	Live learning	C: no intervention (usual care)	Interactive workshops: Workshop 1: core communication skills in healthcare (engaging; empathizing; educating patients of diagnosis, prognosis, and treatment; and enlisting patients in mutually agreed upon treatment plans). Workshop 2: patient adherence, enhancing patients’ health lifestyles, reducing health risk behaviors, and building confidence and conviction in patients to make healthy behavior changes. Workshop 3: sources and nature of interpersonal difficulties between clinicians and patients, recognizing and assessing tension in relationships, acknowledging problems, discovering meaning, showing compassion, setting boundaries, and helping patients find additional support Clinical setting: not specified	Global ratings of the physician-patient interaction Patient satisfaction	4E model: Engage, Empathize, Educate, and Enlist (Keller and Carroll, 1994)
		I: Arm A : doctor workshop + no pt activation Arm B: Pt + Dr intervention Arm C: pt activation, dr not trained			
Helitzer (2011) [35] USA	Live learning	C: no intervention (usual care)	One full-day training: Role-play and training in pt-centred communication skills and elements of motivational training. Individualized feedback on audio + video-taped interactions with simulation patients Clinical setting: adverse childhood events	Effectiveness of training: patient centeredness using RIAS coding scheme	Not specified
		I: One full day training and role play + audiotaped interactions with simulation patients			
Krones (2008) [36, 70] Germany	Live learning	C: Seminars on defined alternative topics that would not interfere with CVD prevention.	Two **interactive** CME sessions and a booklet, a paper-based risk calculator, and individual summary sheets for each patient. CME sessions comprised: epidemiological background of global CVD risk. calculation and ethics of shared decision making practical communication strategies and materials to be applied during consultation. Physicians were taught to calculate and show the effect of several preventive measures simultaneously. **Role play**: Using the script-like decision aid was practiced. Participants received feedback from peers in their groups. Clinical setting: cardiovascular disease	Decisional regret SDM-Q9 Patient participation scale: physician version	The ARRIB-Herz decision aid was developed based on the CREDIBLE criteria
		I: Educational meeting of 2 h with role-play about the DA and SDM			
Kunneman (2020) [50] USA	Online	C: no intervention (usual care)	Minimal training: Participating clinicians at each site completed a training session with a study coordinator, including an overview of the Anticoagulation Choice Shared Decision Making tool and a video tutorial about its intended use. Clinical setting: cardiovascular disease	OPTION12 Decisional conflict Physician satisfaction with consultation	Not specified
		I: Clinicians at each site completed a training session, including an overview of the Anticoagulation Choice Shared Decision Making tool and a video tutorial about its intended use. Very minimal training			
Kunneman (2022) [28] USA	Live learning	C: Usual care consisted of clinicians engaging with their patients as usual with an increased awareness of diabetes care guidelines.	Clinicians received training on how to use the conversation aid during a 10 min group session (including rationale, demonstration of use, role playing), by accessing an online demonstration and a onepage storyboard, and by requesting ad-hoc, one-on-one training during the study. Clinical setting: Diabetes and statins (CV prevention)	Patient satisfaction with consultation Physician satisfaction with consultation	RE-AIM framework: This framework has been developed specifically to address how an intervention, in this case use of decision aids, is implemented in a real-world setting
		I: The use of the Diabetes medication Choice DA during the clinical encounter. Training was provided to clinicians how to use the DA in a group session with role-play.			
Légaré (2012) [56, 57, 71] Canada	Blended	C: no intervention (usual care)	Online self-tutorial + interactive workshop Both the online tutorial and workshop included videos, exercises and decision aids to help physicians communicate to their patients the probability of a bacterial acute respiratory infection and the benefits and harms associated with the use of antibiotics. Clinical setting: respiratory tract infections	Decisional regret Decisional conflict Quality of life Option scale – physician reported Option scale – patient reported Clinicians’ intention to engage in SDM Patient intention to engage in SDM	the Theory of Planned Behaviour
		I: DECISION + 2 consisted of a 2-hour online tutorial followed by a 2-hour on-site interactive workshop			
Loh (2007) [37] Germany	Live learning	C: no intervention (usual care)	Physicians in the intervention group completed modules which included specialized lectures with accompanying questions and discussion rounds, facilitation practice, role-playing, and video exemplars of high-quality shared decision making. Standardized case vignettes and case studies from the general practice were used. All intervention physicians were given decision aids and patient information leaflets for dissemination to the patients. The decision aid was used during the decision-making consultation. Clinical setting: depression	Patients’ Perceived Involvement in Care Patient satisfaction Quality of life	Based on the work of Towle and Godolphin and Elwyn and colleagues
		I: Workshop and role-play for physicians Patients: DA and patient information leaflet			
Mathers (2012) [53] UK	Online	C: no intervention (usual care)	Training session on how to use the PANDA decision aid. The training topics covered included the principles of shared decision making, the importance and clinical effectiveness of DA’s, the evidence for various treatment options for poorly controlled T2DM and essential skills. in risk communication. Clinical setting: diabetes	Decisional conflict Control preference scale	UKMRC framework
		I: Patient DA + healthcare professional training workshop			
Moral (2001) [38] UK	Live learning	C: no intervention (usual care)	Watching **exemplary videos**. We also included information about the scientific evidence of the effectiveness of the diverse communication strategies usually employed. They **practised communication skills** for: (i) establishing an effective relationship; (ii) obtaining biopsychosocial information; (iii) giving information and negotiating; and (iv) closing the interview. **Role-playing, selected video-recorded interviews** as models and as a ‘trigger’, and feedback. Finally, an **interview with a ‘simulated’ patient** was carried out by all doctors, following **individualized feedback** by the facilitator. Clinical setting: musculoskeletal pain / fibromyalgia	GATHARES-CP questionnaire	Not specified
		I: Skills training with role play			
Ngu Haidee (2022) [39] Singapore	Live learning	C: no intervention (usual care)	face-to-face training sessions conducted by two urologists and a PCP. Training 1: the use of VAUS Training 2: covered the nine elements in the SDMQ-9 questionnaire Group discussion about common pitfalls SDM Magic training video was demonstrated Training consultation with a simulated patient + individual feedback Clinical setting: prostatism	SDMQ-9 patient SDMQ-9 physician	Not specified
		I: Physicians: 2 face to face training sessions on SDM and use of the VAUS tool Patients: VAUS DA			
Price Hay-wood (2014) [58] USA	Blended	C: Audit and feedback only	One-on-one 30-minute academic detailing with a physician/communication expert to review ACS guidelines, clinical red flags for identifying patients with low HL, and strategies for effective counseling about cancer screening. Intervention physicians are directed to WebSP10 (web-based service for SP event management) to review SP ratings of their communication skills. Clinical setting: screening	Perceived Involvement in Care	Charles’ and Braddock’s SDM models
		I: Communication skills training for physicians with web-based service + audit and feedback			
Roter (2012) [54] USA	Online	C: Patients received educational materials ! all physicians received the training intervention	E-learning: Separate interactive video glossaries demonstrating communication skills. The clinician glossary included 288 skill clips demonstrating the 23 physician skills. The skill demonstrations were scripted and filmed using a total of 8 actors portraying patients and health care providers of diverse age, ethnicity and gender. Clinical setting: cardiovascular disease	Patient satisfaction with consultation	LEAPS: LISTEN, EDUCATE, ASSESS, PARTNER AND SUPPORT
		I: Patients and physician web-based tool			
Sanders (2017) [29] The Netherlands	Live learning	C: no intervention (usual care)	Small groups and 2 training sessions that focused on the SDM process and evidence-based treatment of low back pain. In the training sessions, **group discussion**, **theory**, **role-playing** and **reflections** on personal behavior were alternated To stimulate their use of SDM skills during the actual consultations, we provided the GPs with a desktop tool containing group-formulated open-ended questions applicable to the consecutive SDM process elements and standard sentences that could be used to positively reinforce patients’ treatment expectations Clinical setting: low back pain	OPTION scale	The training was based on the learning principles described by Kolb and the SDM behavioural process elements developed by Elwyn.
		I: Training sessions on SDM in low back pain, feedback on videotaped consultation			
Sepucha (2022) [51] USA	Online	C: no intervention (usual care)	Online: case studies and interactive exercises to simulate conversations with older patients about CRC testing. There was the ability to submit cases and get feedback from study investigators There was an opportunity to complete an additional telephone-based simulated patient interaction to practice skills. Clinical setting: screening	Patient satisfaction with consultation Shared Decision-Making Process Scale	Not specified
		I: a 2 h online SDM training course			
Stewart (2007) [40] Canada	Live learning	C: Traditional 2-hour CME showing a videotaped consultation, which was then discussed	(1) literature—a description of the benefits of improved patient-physician communication for both patients and doctors (2) physicians’ perspectives—participating physicians ventilated about barriers to and shared solutions for effective communication (3) patients’ perspectives—first, a videotape of the findings of the qualitative study of breast cancer patients’ issues regarding communication, and second, breast cancer survivors in person talking about their own concerns (4) video demonstration—a scripted “not-so-good” and “better” interaction between a breast cancer patient/actress and physician (5) practice with standardized patients and videotape review with feedback. Clinical setting: screening	Quality of life Patient satisfaction Objective patient-centred communication measure Patient perceptions of patient-centeredness	Conceptual framework for patient-centered communication
		I: a new 6-hour CME including the traditional CME plus 2 new elements: a discussion of the patients’ perspectives, and a tape review with individual feedback.			
Tai-Seale (2016) [41] USA	Live learning	C: no intervention (usual care)	They developed an intervention, called Open Communication (OpenComm). The first element of this intervention was a 2-minute animated video, developed to illustrate open communication behaviors for patients and primary care providers. The video normalized setting a joint agenda, asking questions, and requesting information on other options. A standardized patient instructor provided individualized communication coaching for primary care providers. During the interval between the first and second standardized patient instructor coaching sessions, a “practice patient” from among that provider’s regularly scheduled patients was recruited to use the intervention materials in his or her visit. Clinical setting: not specified	OPTION-5 scale	Four Habits Model: Invest in the beginning, elicit the patient’s preferences, demonstrate empathy, and invest in the end.
		I: OpenComm			
Tilburgs (2020) [42] The Netherlands	Live learning	C: no intervention (usual care)	Practice ACP conversations with training actors. To structure ACP conversations, a model for shared decision making with older adults with frailty was introduced. Clinical setting: advanced care planning	Quality of life CollaboRATE scale	The intervention was developed according to the adapted framework of the Medical Research Council Guidance for the development and evaluation of complex interventions. The SDM model used consists of 6 steps including the traditional steps of choice talk, option talk, and decision talk.
		I: SDM training for GPs in advanced care planning			
Tinsel (2013) [43] Germany	Live learning	C: no intervention (usual care)	The training program includes following elements: (1) information on arterial hypertension (2) physician-patient communication and risk communication (3) the process steps of SDM (4) motivational interviewing (5) introduction of a decision table listing options to lower CVR and (6) use of case vignettes for role plays simulating physician-patient consultations. Clinical setting: cardiovascular disease	SDM-Q9	Not specified
		I: SDM training for GPs			
Wilkes (2013) [52] USA	Online	C: no intervention (usual care)	Web-based 30-minute tool on prostate cancer screening: importance of prostate cancer in men’s health, limitations of screening, the central importance of each individual’s values and preferences. Laminated screen shots of essential diagrams to physicians for use while counseling patients about likelihoods of harm and benefit around prostate cancer screening. Clinical setting: cancer screening	Patient satisfaction with consultation Kaplan’s validated shared decision-making instrument Physicians’ perception of SDM	Not specified
		Arm A: web based tool for physicians. Arm B: web based tool for physicians + activated patients through web based tool			
Wollny (2021) [44] Germany	Live learning	C: no intervention (usual care)	GPs specially trained in patient-centered communication visited enrolled GPs. This peer-visit aimed at sensitizing for patients’ concepts of disease and their views, attitudes, and behaviors by using patient centered communication. Next, GPs were encouraged to use the electronic decision-aid to increase shared decision making. Enrolled physicians were offered a workshop on patient-centered communication Clinical setting: diabetes	SDM-Q9	Not specified
		I: GPs were trained in patient-centered communication and the use of the Arriba DA			

### Risk of bias of included studies

**Fig. 2:**
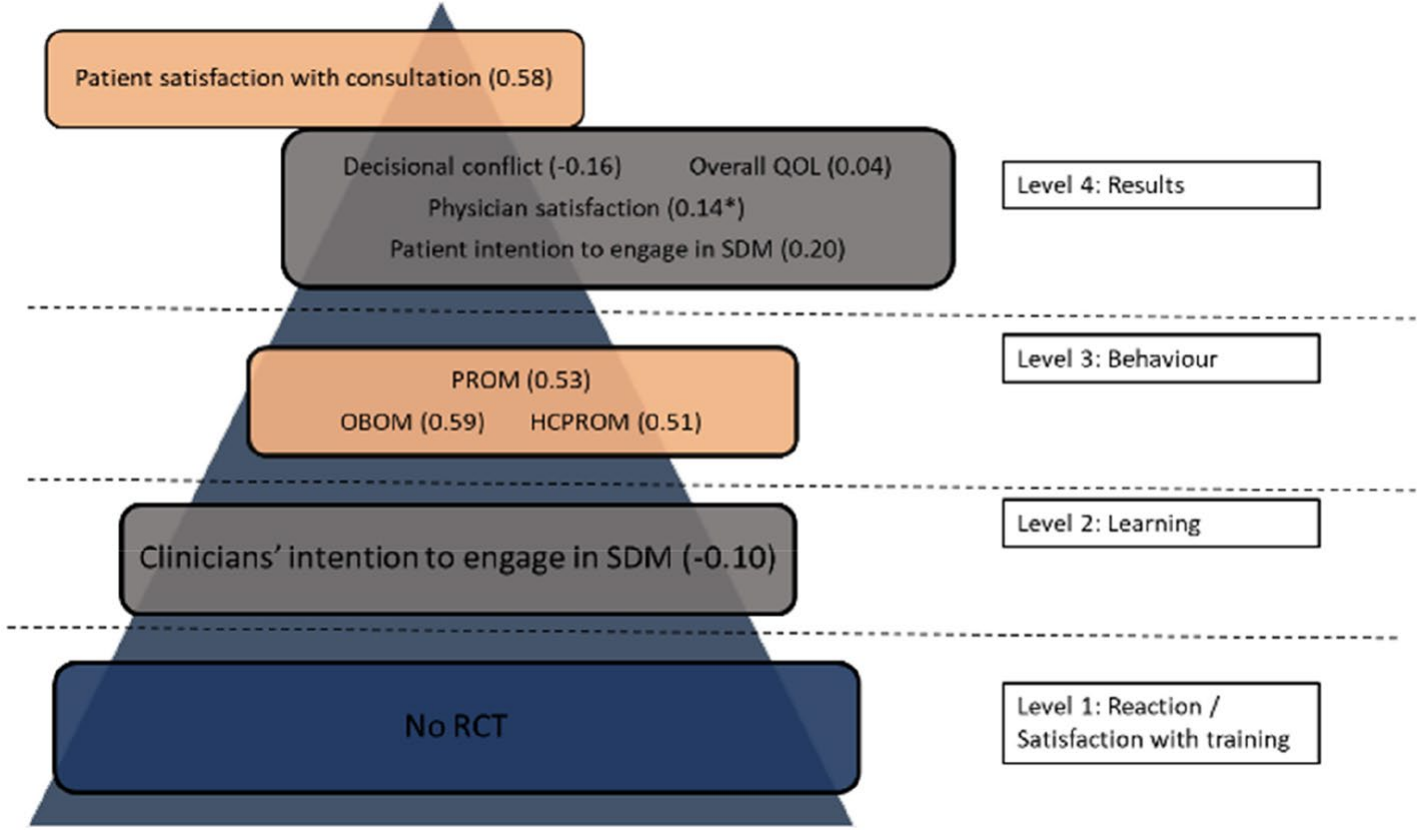
Outcome measures of all SDM training programs (online + live + blended) categorized by Kirkpatrick level. Numbers in brackets are standardized mean difference, numbers with * indicate a risk difference. Color legend: blue = no studies. Grey = small effect size (Cohen’s d < 0.2). Orange = medium effect size (Cohen’s d 0.2–0.5). Green: large effect size (Cohen’s d > 0.8). RCT = randomized controlled trial. SDM = Shared Decision making. PROM = patient reported outcome measure. OBOM = observer reported outcome measure. HCPROM = healthcare professional reported outcome measure. QOL = quality of life

All studies were rated as having a low risk of bias for Sect. 1 on clear aims and justification (Fig. 2). A low risk of bias was rated for Sect. 2 in 16 studies (55%), for Sect. 3 in 8 studies (27%), for Sect. 4 in 19 studies (65%), for Sect. 5 in 19 studies (65%), for Sect. 6 in 17 studies (59%) and for Sect. 7 in 6 studies (21%). A more detailed description of the risk of bias can be found in Additional file 6.

#### Overview of training programs included in the review

We found a broad variety of training content for teaching SDM skills to general practitioners. We categorized the training formats into three groups: online learning [45–54], live learning [28–44] and blended learning [55, 56]. For the online programs, training content varied from a 15 min video based training package on how to use a decision aid and apply SDM [45], to a web-based tool of several hours where participants could review a recorded consultation with a simulation patient with personalized feedback and where illustrative video vignettes could be found [47]. For the live learning programs, there was also a variety of training durations (2 h workshops [33] versus workshops over several days [39]). Most training programs used role-plays [33, 35–37] or simulation patients [31, 32] to practice SDM skills. Two studies also integrated a communication expert in their training [31, 44]. We found two studies that offered very minimal training were participants gathered in a meeting and they were trained in how to use a decision aid [46, 50]. For the two blended learning programs, one program offered the participants strategies for effective counseling about cancer screening with a physician and communication expert and a web-based tool to review individualized feedback on communication skills [55]. The second training program offered an online self-tutorial with an interactive workshop and exercises [56]. A more detailed description of the training programs can be found in Table 1 at the end of the review.

#### Effectiveness of SDM training based on Kirkpatrick model

We summarized the outcome measures of all studies, and categorized these outcomes according to the Kirkpatrick model (Fig. 2). Forest plots of each Kirkpatrick level separate can be found in Additional file 2. When an outcome was presented both in a continuous and categorical scale, we categorized only the continuous outcome according to the Kirkpatrick model. An overview of the outcomes used in every study can be found in Table 1.

### Kirkpatrick level 2

#### Clinicians’ intention to engage in SDM

Only one study [57] reported on clinicians’ and patients’ intention to engage in SDM showing little or no difference between groups (Mean difference (MD) -0.10, 95% CI -0.29–0.09) *(*Additional file A2-1).

### Kirkpatrick level 3

#### Observer reported outcome measure

**Fig. 3:**
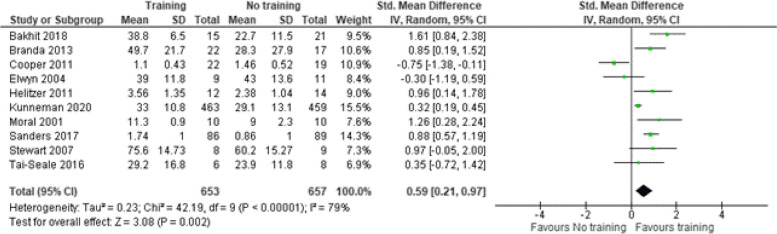
Shared decision making skills – Observer reported scales. SD = standard deviation. Std. mean difference = standardized mean difference. IV = inverse variance. 95% CI = 95% confidence interval

Ten studies [29, 32, 35, 38, 40, 41, 45–47, 50] used an observer reported (OBOM) SDM scale to measure SDM from an observer’s perspective (Fig. 3). The OBOMS used were the OPTION-12 scale, patient centeredness using RIAS, the GATHARES-CP questionnaire and a patient-physician communication behaviour scale. The estimate of the standardized mean difference (SMD) was 0.59 (95% CI 0.21–0.97) indicating a medium-large effect of the intervention.

#### Patient reported outcome measure

**Fig. 4:**
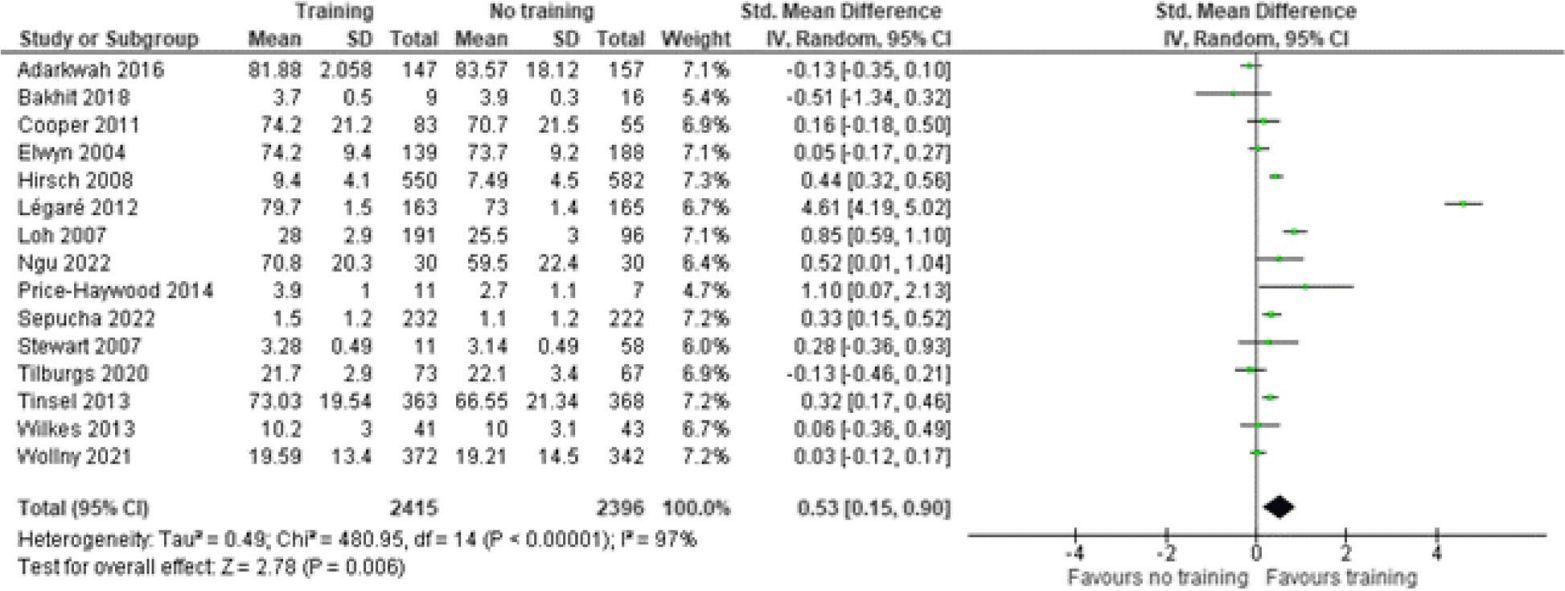
Shared decision making skills – Patient reported scales. SD = standard deviation. Std. mean difference = standardized mean difference. IV = inverse variance. 95% CI = 95% confidence interval

Fifteen studies [30, 32, 36, 37, 39, 40, 42–45, 47, 51, 52, 57, 58] used a patient-reported outcome measure (PROMs) to measure SDM from a patient perspective (Fig. 4). These PROMs were an SDM process scale, patients’ perception of SDM scale, patient ratings of their clinicians participatory decision-making skills, the control preference scale, CollaboRATE scale, SDM-Q9, Comrade scale, patient perception of patient-centeredness scale and patient perceived involvement in care. The SMD was 0.53 (95% CI 0.15–0.90) indicating a medium effect of the intervention.

#### Healthcare professional reported outcome measure

**Fig. 5:**
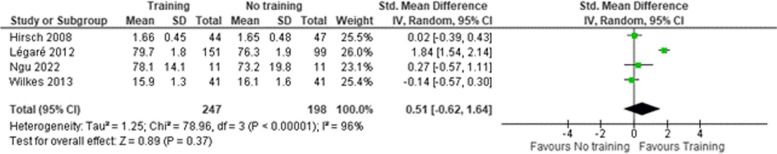
Shared decision making skills – Healthcare professional reported scales. SD = standard deviation. Std. mean difference = standardized mean difference. IV = inverse variance. 95% CI = 95% confidence interval

Four studies [36, 39, 52, 57] used a healthcare professional-reported (HCPROM) scale to measure SDM from a clinician perspective (Fig. 5). These HCPROMs were an SDM adapted Kaplan instrument which included perception of own SDM skills, SDM-Q9 doc and an adjusted patient participation scale. The SMD was 0.51 (95% CI -0.62–1.64) indicating a medium effect of the intervention.

### Kirkpatrick level 4

#### Patient satisfaction with consultation

Four studies [37, 40, 52, 54] reported patient satisfaction with consultation. The SMD was 0.58 (95% CI 0.03– 1.12) indicating a medium-large effect of the intervention (Additional file A2-2 and A2-3).

#### Physician satisfaction with consultation

Two studies [28, 50] reported physician satisfaction with consultation. The risk difference was 0.14 (95% CI -0.10–0.39), indicating a small effect of the intervention (Additional file A2-4).

#### Decisional regret

Two studies [36, 57] reported on decisional regret. The SMD was 0.13 (95% CI -0.16–0.42), indicating a small effect in favor of no training intervention (Additional file A2-5).

#### Decisional conflict

**Fig. 6:**
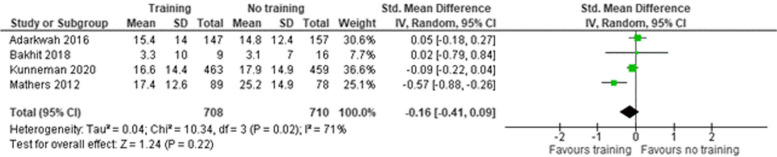
Decisional conflict. SD = standard deviation. Std. mean difference = standardized mean difference. IV = inverse variance. 95% CI = 95% confidence interval

Four studies [30, 45, 50, 53] reported on decisional conflict (Fig. 6). The SMD was − 0.16 (95% CI -0.41–0.09) indicating that the intervention had a small effect.

#### Patients’ intention to engage in SDM

Only one study [57] reported on patients’ intention to engage in SDM (Additional file A2-6). The MD was 0.20 (95% CI -0.05–0.45), indicating a small effect of the intervention.

#### Quality of life

Six studies reported quality of life (QOL): five studies [32, 37, 40, 47, 57] reported a mental health scale, three studies [32, 47, 57] reported a physical health scale and one study [42] reported an overall QOL scale. The SMD for the mental health scale was 0.05 (95% CI -0.08–0.18), for the physical health scale 0.08 (95% CI -0.06–0.22) and for the overall QOL scale was 0.04 (95% CI -0.29–0.37), all indicating a very small effect of the intervention on QOL (Additional file A2-7).

### Effectiveness of SDM training based on training format

Additionally, we categorized the studies based on training format (online, live or blended learning) and further categorized the outcome measures according to the Kirkpatrick model. Forest plots of each outcome measure separate can be found in Additional files 4, 5 and 6.

#### Online learning

**Fig. 7:**
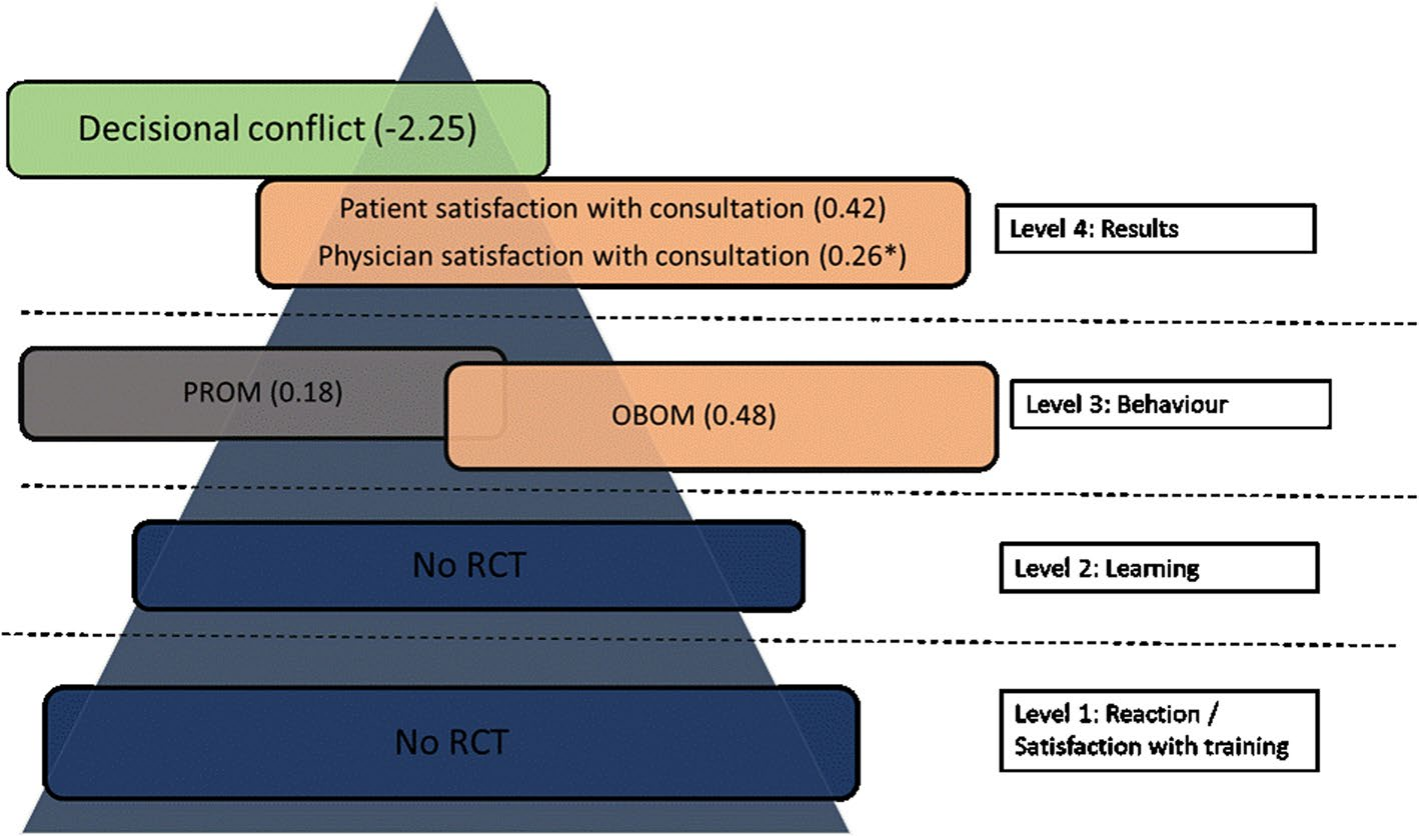
Outcome measures of online training programs based on Kirkpatrick level. Numbers in brackets are standardized mean difference, numbers with * indicate a risk difference. Color legend: blue = no studies. Grey = small effect size (Cohen’s d < 0.2). Orange = medium effect size (Cohen’s d 0.2–0.5). Green: large effect size (Cohen’s d > 0.8). RCT = randomized controlled trial. SDM = Shared Decision making. PROM = patient reported outcome measure. OBOM = observer reported outcome measure. HCPROM = healthcare professional reported outcome measure. QOL = quality of life

A total of ten studies [45–54] reported an online SDM training program. Six studies [45, 46, 48, 50–52] compared training interventions targeting general practitioners with usual care (no training intervention). One study [49] compared training interventions targeting general practitioners with another training intervention (e.g. GPs who received a brochure). In addition to the GP-directed intervention, three other studies [47, 53, 54] also compared patient-directed interventions (patient decision aid, patient activation or patient educational materials) with other interventions targeting patients and GPs. Figure 7 summarizes the outcome measures for every Kirkpatrick level.

##### Patient reported outcome measure

**Fig. 8:**
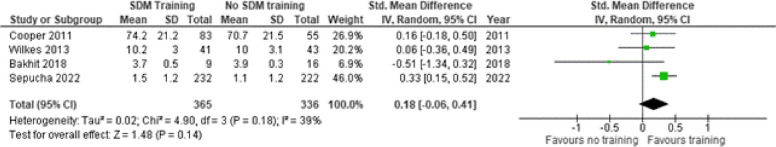
Shared decision making skills – Patient reported scales. SD = standard deviation. Std. mean difference = standardized mean difference. IV = inverse variance. 95% CI = 95% confidence interval

Four studies [45, 47, 51, 52] used a PROM to measure the use of SDM from a patient’s perspective (Fig. 8). The SMD was 0.18 (95% CI -0.06–0.41) indicating a small effect of the intervention.

##### Healthcare professional reported outcome measure

Only one study [52] reported a HCPROM (adapted Kaplan instrument which included perception of own SDM skills). The mean difference was − 0.20 indicating no effect of the intervention (Additional file A3-2).

##### Observer reported outcome measure

**Fig. 9:**
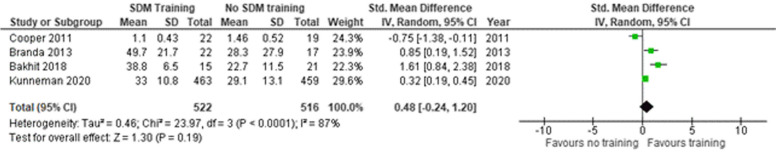
Shared decision making skills – Observer reported scales. SD = standard deviation. Std. mean difference = standardized mean difference. IV = inverse variance. 95% CI = 95% confidence interval

Five studies [45–47, 49, 50] reported OBOMs (Fig. 9). One study [49] was excluded from the analysis due to missing SD. The SMD was 0.48 (95% CI -0.24–1.20) indicating a medium effect of the intervention.

##### Patient satisfaction with consultation

Two studies [52, 54] reported on patient satisfaction with consultation. The SMD was 0.42 (95% CI -0.42–1.25) indicating a medium effect of the intervention (Additional file A3-3 and A3-4).

##### Physician satisfaction with consultation

Only one study [50] reported physician satisfaction with consultation. The risk difference was 0.26 (95% CI 0.21–032) in favor of the intervention (Additional file A3-5).

##### Decisional conflict

**Fig. 10:**
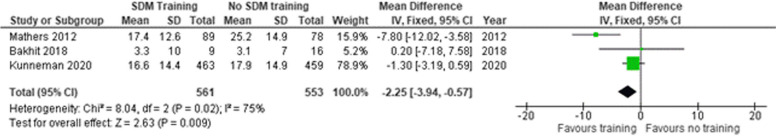
Decisional conflict. SD = standard deviation. Std. mean difference = standardized mean difference. IV = inverse variance. 95% CI = 95% confidence interval

Three studies [45, 50, 53] reported on decisional conflict using the decisional conflict scale (Fig. 10). The mean difference was − 2.25 (95% CI -3.94 – -0.57 ) indicating a large effect of the intervention.

#### Live learning

**Fig. 11:**
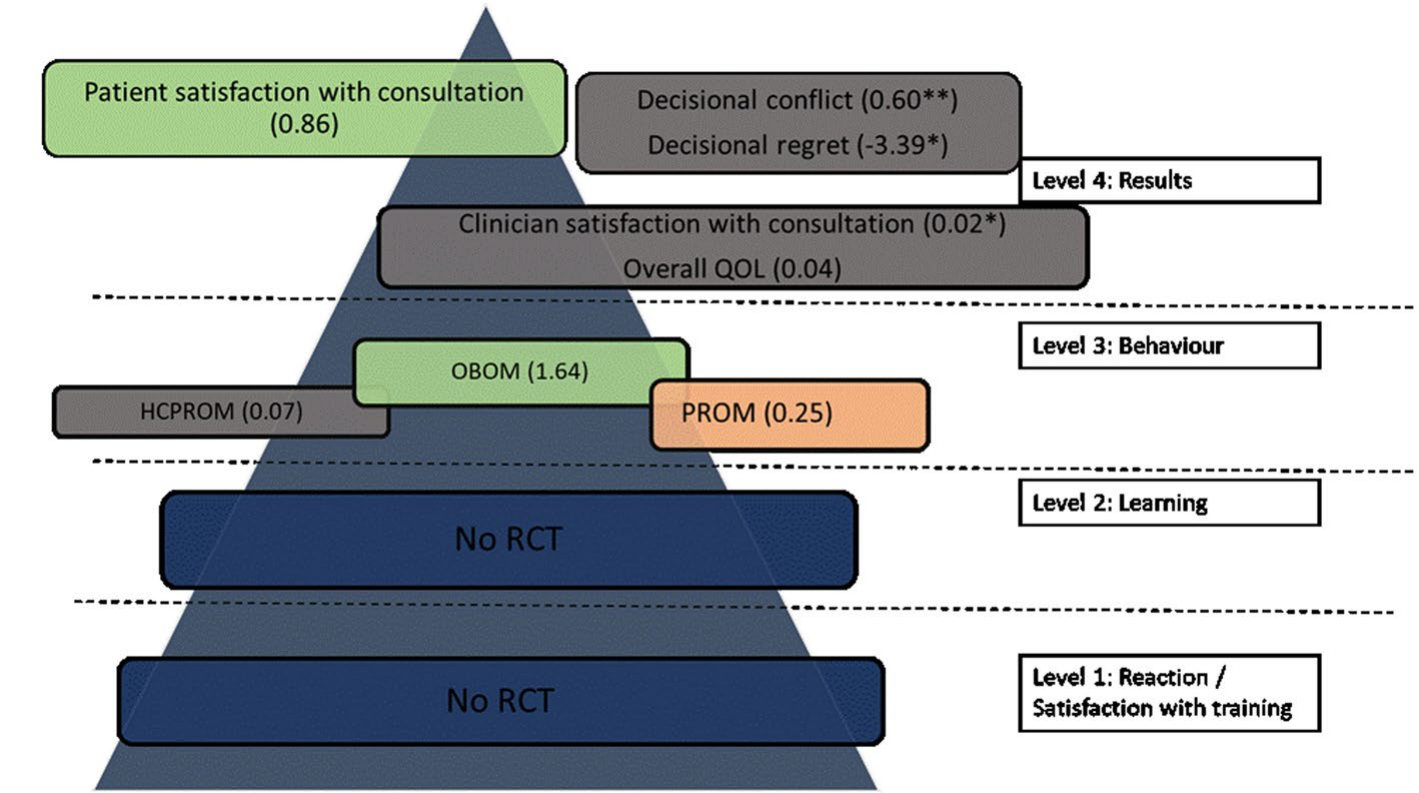
Outcome measures of live training programs based on Kirkpatrick level. Numbers in brackets are standardized mean difference. Numbers with * indicate a risk difference. Numbers with ** indicate the mean difference. Color legend: blue = no studies. Grey = small effect size (Cohen’s d < 0.2). Orange = medium effect size (Cohen’s d 0.2–0.5). Green: large effect size. RCT = randomized controlled trial. SDM = Shared Decision making. PROM = patient reported outcome measure. OBOM = observer reported outcome measure. HCPROM = healthcare professional reported outcome measure. QOL = quality of life. (Cohen’s d > 0.8)

A total of 17 studies [28–44] reported on a live SDM training program. Ten studies [29, 31, 34–36, 38, 41–44] compared training interventions targeting GPs with usual care (no training intervention). Three studies [28, 32, 40] compared training interventions targeting GPs with another training intervention (risk communication workshop, increased awareness of diabetes care guidelines, traditional CME with feedback on taped consultations). Four studies [30, 33, 37, 39] also compared, next to the GP directed intervention, patient-directed interventions (patient decision aid, patient activation or patient educational materials) with other interventions targeting patients and GPs. Figure 11 summarizes the outcome measures for every Kirkpatrick level. A unit of analysis error was observed in one study, and so we could not estimate the effect size [34].

##### Patient reported outcome measure

**Fig. 12:**
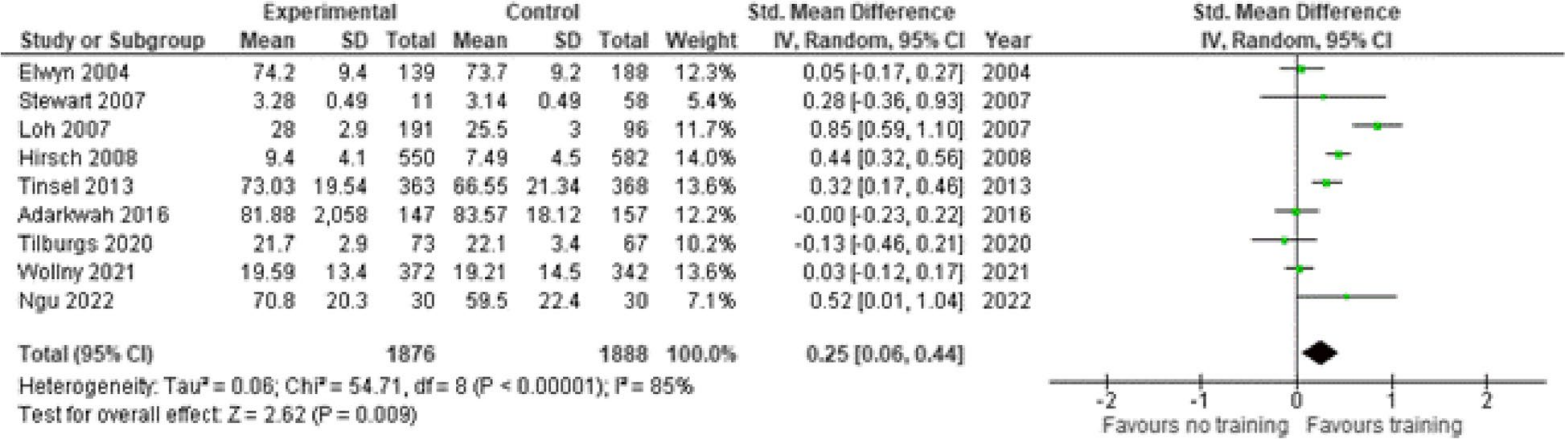
Shared decision making skills – Patient reported scales. SD = standard deviation. Std. mean difference = standardized mean difference. IV = inverse variance. 95% CI = 95% confidence interval

A total of nine studies [30, 32, 36, 37, 39, 40, 42–44] reported PROMs (Fig. 12). The SMD was 0.25 (95% CI 0.06–0.44) indicating a medium effect of the intervention (Additional file A4-1).

##### Observer reported outcome measure

**Fig. 13:**
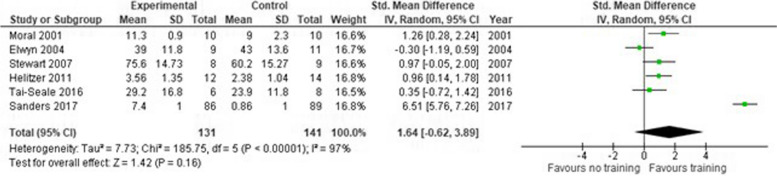
Shared decision making skills – Observer reported scales. SD = standard deviation. Std. mean difference = standardized mean difference. IV = inverse variance. 95% CI = 95% confidence interval

Six studies [29, 32, 35, 38, 40, 41] reported OBOMs (Fig. 13). The SMD was 1.64 (95% CI -0.62–3.89) indicating a high effect of the intervention. One RCT [33] was excluded from analysis due to missing mean and SD of the control group.

##### Healthcare professional reported outcome measure

Two studies [36, 39] reported HCPROMs. The SMD was 0.07 (95% CI -0.30–0.44) indicating a very small effect of the intervention (Additional file A4-2).

##### Patient satisfaction with consultation

**Fig. 14:**
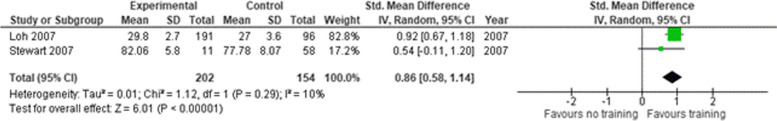
Patient satisfaction with consultation. SD = standard deviation. Std. mean difference = standardized mean difference. IV = inverse variance. 95% CI = 95% confidence interval

Two studies [37, 40] reported patient satisfaction with the consultation (Fig. 14). The SMD was 0.86 (95% CI 0.58–1.14) indicating a high effect of the intervention (Additional file A4-3).

##### Decisional regret

Only one study [36] reported decisional regret finding no or little difference between groups (mean difference − 3.39, 95% CI -56.22–49.44) (Additional file A4-4).

##### Decisional conflict

One study [30] reported on decisional conflict. The mean difference was 0.60 (95% CI -2.38–3.58) indicating little difference between the control and intervention group Additional file A4-5).

##### Clinician satisfaction with consultation

One study [28] estimate of the RD was 0.02 (95% CI: -0.05–0.10) indicating that the intervention may have made little or no difference increasing clinician satisfaction with consultation (Additional file A4-6).

##### Quality of life

Four studies reported QOL: three studies [32, 37, 40] reported a mental health scale, one study [32] reported a physical health scale and another one study [42] reported an overall QOL scale. The SMD for overall QOL 0.04 (95% CI -0.10–0.19) indicating a small effect of the intervention (Additional file A4-7).

#### Blended learning

**Fig. 15:**
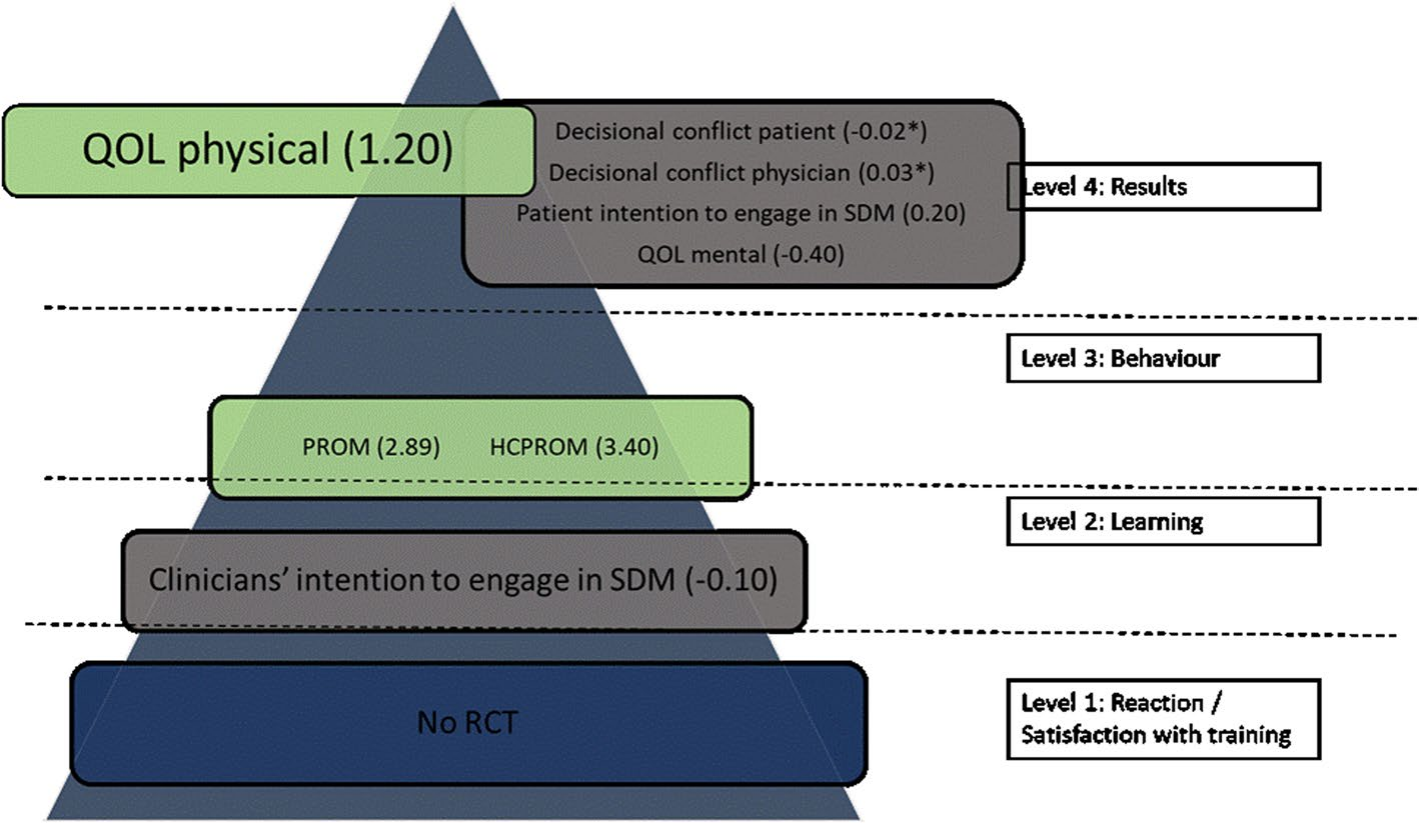
Outcome measures of blended training programs based on Kirkpatrick level. Numbers in brackets are standardized mean difference, numbers with * indicate a risk difference. Color legend: blue = no studies. Grey = small effect size (Cohen’s d < 0.2). Orange = medium effect size (Cohen’s d 0.2–0.5). Green: large effect size (Cohen’s d > 0.8). RCT = randomized controlled trial. SDM = Shared Decision making. PROM = patient reported outcome measure. OBOM = observer reported outcome measure. HCPROM = healthcare professional reported outcome measure. QOL = quality of life. (Cohen’s d > 0.8)

Two studies reported on a blended SDM training program. One study [57] compared training interventions targeting general practitioners with usual care (no training intervention). One study [58] compared training interventions targeting general practitioners with another training intervention (GPs received audit and feedback). Figure 15 summarizes the outcome measures for every Kirkpatrick level.

##### Clinicians’ intention to engage in SDM

Only one study [57] reported on clinicians’ intention to engage in SDM showing little or no difference between groups (mean difference − 0.10; 95% CI -0.26–0.09) (Additional file A5-1).

##### Patient reported outcome measure

**Fig. 16:**
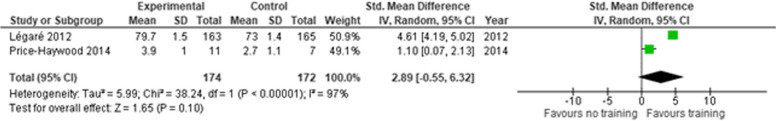
Shared decision making skills –Patient reported scales. SD = standard deviation. Std. mean difference = standardized mean difference. IV = inverse variance. 95% CI = 95% confidence interval

Both studies reported PROMs (Fig. 16). The SMD was 2.89 (95% CI -0.55–6.32 ) indicating a large effect of the intervention (Additional file A5-2).

##### Healthcare professional reported outcome measure

Only one study [57] reported on HCPROM for SDM skills with a mean difference of 3.40 (95% CI 2.93–3.87) indicating a large effect (Additional file A5-3).

##### Decisional regret

Only one study [57] reported on decisional regret, with an MD of 4.80 (95% CI 1.20–8.40) in favor of no intervention (Additional file A5-4).

##### Decisional conflict

Only one study [57] reported on decisional conflict using a categorical decisional conflict scale in view of the patient and the physician showing little or no difference between groups (RDs of -0.02 and 0.03 respectively) (Additional file A5-5 and A5-6).

##### Patients’ intention to engage in SDM

Only one study [57] reported on patients’ intention to engage in SDM. The MD was 0.20 (95% CI -0.05–0.45) indicating a small effect of the intervention (Additional file A5-7).

##### Quality of life

Only one study [57] reported on QOL with a mean difference of 1.20 (95% CI -0.38–2.78) for the physical subscale and − 0.40 (95% CI -2.23–1.43) for the mental subscale indicating a large effect for the physical health scale and low effect for the mental health scale (Additional file A5-8).

## Discussion

To our knowledge, this is the first systematic review on the effectiveness of an SDM training program for GPs using the Kirkpatrick evaluation model. We also considered the effect of an SDM training program based on the training format (online, live or blended learning). For our first research question, we found a broad variety of training programs for GPs (Table 1). They vary in training format (online, live, blended), targeted population (GPs alone or combined with patient interventions), duration (several hours to several days) and intensity (demonstrating how to use a decision aid to role-playing with actors). Concerning our second research question, we observed that a training program for GPs has a medium impact on PROMs, OBOMs, HCPROMs and patient satisfaction with consultation. We observed only a small effect for the outcomes clinicians’ intention to engage in SDM, decisional conflict, QOL, physician satisfaction and patients’ intention to engage in SDM. For our third research question, we observed that online training programs have a strong effect on decisional conflict and a medium effect on OBOM. For live training, we found a high effect on patient satisfaction and OBOM and a medium effect PROM. For blended training programs, we found a high impact for physical QOL, PROM and HCPROM. We can conclude that there is a slight preference for blended learning, however one should keep in mind we found only two studies with this training format.

There are a few important lessons to consider after conducting this review. We found numerous instruments for measuring SDM skills confirming there is still no standardized instrument for assessing the effect and use of SDM by GPs. Until now, there is still no consensus on which type of measurement is most suitable and accurate for SDM indicating how challenging research on SDM training effectiveness is. The most commonly used OBOM instrument was the OPTION-12 scale [59]. This validated scale measures SDM skills of healthcare professionals on 12 items such as “The clinician draws attention to an identified problem as one that requires a decision making process”, “The clinician lists options, which can include the choice of ‘no action’, “The clinician explains the pros and cons of options to the patient” and “The clinician elicits the patient’s preferred level of involvement in decision-making” on a scale of 0 to 100 where a higher score indicates more SDM. It is important to note that almost all studies report an overall OPTION score meaning that if (no) improvement in SDM skills is observed after a training intervention, one cannot explain which element of these 12 items is improved or need more attention [60, 61]. It would therefore be interesting to explore which items need more attention in an SDM training program, which may improve its effectiveness on acquired SDM skills. Shared decision making is a process and not all SDM elements should be covered in one consultation which makes it more complicated to assess SDM skills. Since most studies used SDM scales based on one consultation, it is possible the SDM skills of the healthcare professional are underestimated. It would therefore be interesting to conduct more controlled before-after studies to assess the effect of a training intervention versus no intervention and to evaluate SDM skills over time to assess its sustainability. This confirms that measurement of SDM and an appropriate study design need improvement. Furthermore, one should keep in mind that SDM should be taught in a more flexible approach. Hence SDM is patient-centered, it does not mean it always have to be patient driven. The use of SDM should be flexible according to the patients’ preferences for involvement but also to patient characteristics like race and belief [62]. At last, it is important to note that we relied on published material regarding the content of the training program. It is not clear when and why authors defined their training program as a true SDM training program. Until now, there is no consensus about the set of SDM core competencies [21]. One group of SDM experts and patient representatives did define 2 types of competencies physicians should acquire to help their patients to be involved in the decision making process, being relational competencies and risk communication competencies [21]. It is not clear whether training programs included in our review developed and evaluated after 2014 relied on these two core competencies. Where needed, we contacted authors of included studies but were not always able to get an answer from them. This may demonstrate a lack of transparency in the content and evaluation of the SDM training research field.

We found no other review assessing the effectiveness of an SDM training program for GPs. An uncertain effect of interventions for increasing the use of SDM by healthcare professionals has been reported previously [63]. In this review, the authors divided the interventions based on the targeted population (healthcare professional, patients or both) which we did not to keep the number of studies per outcome as high as possible. Specifically for primary care, the use of SDM reduced antibiotic prescriptions for acute respiratory tract infections without decreasing patient satisfaction with consultation [64]. However, there is no report of acquired SDM skills of the healthcare professionals after the training in this review. Furthermore, no important benefits for health professionals’ skills, knowledge or patient outcomes from e-learning compared to traditional learning have been reported [65]. E-learning programs may be a better choice when the aim is to reach a large number of physicians however, practicing acquired communication skills in real life potentially has a greater impact on improving physicians’ skills. Finally, blended learning compared to traditional learning has a large positive effect on knowledge acquisition among healthcare professionals, probably because participants are able to review electronic materials as often as necessary and at their own pace [66]. Blended learning is considered an upgrade from traditional learning as it combines the advantages of online and live learning [21, 67].

### Strengths and limitations

This study has several strengths. First, we used numerous databases and grey literature to collect as many studies as possible. We conducted a second search after nine months to be sure no new eligible studies were published. Furthermore, we decided to include studies that used different evaluation perspectives to ensure full coverage of SDM outcome measures. To the best of our knowledge, this is the first review comparing acquired SDM skills based on training format. Changes can be made to the training approach (training format) and to training evaluation (what core competencies a training program should contain to be categorized as an SDM training? ).

However, several limitations should be noted. We found a high heterogeneity in multiple studies and outcome measures challenging the interpretation of the reported outcomes. This heterogeneity could be explained by the variety of clinical contexts in which these studies were conducted (respiratory tract infections versus advanced care planning). As previously reported, there is no consensus on which outcome measure is the most accurate for measuring SDM. It is not clear whether one outcome measure should be preferred over the other. Because of the high number of different outcome scales, it is challenging to compare training programs that used different outcome measurements for evaluating SDM skills. It is also important to mention we did not, in contrast to the review of Légaré [63], further divide the interventions based on population targeted (physician directed, patient directed or patient and physician directed). It could be that patient and physician directed interventions have a different/higher impact on SDM outcomes compared to physician-directed interventions alone. The scope of our review is both a strength and a limitation. Not restricting our scope to a specific clinical problem or outcome measure increased the number of studies that could be included. However, restricting to primary care may have caused that possible effective SDM training programs in secondary care have been excluded from this review. Finally, our findings are further limited by inadequate descriptions of the training program in many of the included studies which could also explain the heterogeneity in our results.

It is not possible to draw firm conclusions based on this review (due to heterogeneity of the studies and variety in measurement instruments) regarding the effects of training format on SDM skills and SDM related outcome or how best to design educational meetings specifically for primary care. Nonetheless, we would argue that our review provides a useful context in which to interpret the findings of the individual trials included in this review as well as other studies that address more specific questions about the effects of SDM training interventions.

### Implications for future research and practice

Future reports of trials of SDM training programs should include clear and detailed descriptions of the interventions, including the proportion of the target audience that attended, the teaching techniques, whether there was any skills practice and when/why a training program is defined as being SDM. Whenever possible, cluster randomized designs should be used in combination with process evaluations to further our understanding of why interventions do or do not work and of the variations in their effects. It seems consensus is still lacking concerning the most appropriate training format, as well as the most appropriate measurement instrument(s). The results from this review can assist researchers in comparing different training formats and investigating their effectiveness. We would also recommend to evaluate outcomes measures ranked as high as possible in the Kirkpatrick model since this indicates a higher effectiveness of the training program. One should keep in mind that a ‘perfect in-theory’ SDM training format and program does not always have the desired effect on SDM skills if the targeted population is not interested in SDM. Currently, we are also conducting a qualitative study with GPs (in practice), hospital specialists and residents to assess their learning needs and preferences for an SDM training program to develop an SDM training – complementary to the results found in this review – for healthcare professionals in Belgium.

## Conclusion

Our review demonstrated that SDM training programs improve patient and observer reported SDM skills in GPs and carefully favors a blended training program (regarding Kirkpatrick levels 3 and 4) above an online or live approach. Direct comparisons of different types of educational formats are needed to develop the most appropriate and effective SDM training format. Future research would benefit from less variation in outcome assessments, with a focus on observer and patient reported outcome measures to evaluate the effect of training on acquired SDM skills. To evaluate Kirkpatrick level 4, we believe that patient reported outcomes are most appropriate (satisfaction with consultation, decisional conflict and regret), since these best represent patients’ perception of involvement in the decision making process.

### Supplementary Information

Supplementary Material 1.

Supplementary Material 2.

Supplementary Material 3.

Supplementary Material 4.

Supplementary Material 5.

Supplementary Material 6.

## Data Availability

Availability of data and materials: The datasets used and/or analysed during the current study are available from the corresponding author on reasonable request.
