# Supplementary material for: A systematic review of shared decision making training programs for general practitioners
Source: BMC Med Educ. 2024 May 29;24:592. doi: 10.1186/s12909-024-05557-1 (PMC11137915; doi:10.1186/s12909-024-05557-1)
Supplement: Supplementary file 1 — Supplementary Material 1. [file 12909_2024_5557_MOESM1_ESM.pdf]

Additional file 1: Search strategy

Pubmed Search conducted on 09/12/2022

Search	Query	Results
**#1**	"Physicians"[Mesh:NoExp] OR "General Practitioners"[Mesh] OR "Physicians, Family"[Mesh] OR "General Practice"[Mesh] OR "Physicians, Primary Care"[Mesh] OR “general pract*”[tiab] OR “physician*”[tiab] OR “family doct*”[tiab] OR “general doct*” [tiab] OR “medical pract*”[tiab] OR “family pract*”[tiab] OR “health care provider*”[tiab] OR "healthcare provider*"[tiab] OR "healthcare professional*"[tiab] OR "health care professional*"[tiab] OR “primary care”[tiab]	827.719
**#2**	("Decision Making, Shared"[Mesh] OR "Shared Decision*"[tiab] OR "Sharing Decision*"[tiab] OR "SDM"[tiab] OR "patient decision-making"[tiab] OR "patient decisionmaking"[tiab] OR "participatory decision*"[tiab] OR "Patient-centered"[tiab] OR "Patient-centred"[tiab] OR "person-centered"[tiab] OR "person-centred"[tiab] OR “advanced communicati*”[tiab] OR “informed decision-making”[tiab]) OR (("Physician-Patient Relations"[Mesh] OR “Physician-Patient Relation*”[tiab] OR “Clinician-Patient Relation*”[tiab] OR “Doctor-Patient Relation*”[tiab] OR "Communication"[Mesh:NoExp] OR "Communicat*"[tiab] OR "intercourse*"[tiab] OR “inter course*”[tiab] OR "interchang*"[tiab] OR "inter chang*"[tiab] OR "dialogu*"[tiab] OR "talk*"[tiab] OR "conversation*"[tiab] OR "Patient Participation"[Mesh] OR "participat*"[tiab] OR "involv*"[tiab] OR "Patient-centered care"[Mesh:NoExp] OR “Participative Decision Making”[tiab] OR “patient-focus*”[tiab] OR "engag*"[tiab]) AND ("Decision Making"[Mesh:NoExp] OR "Decision making"[tiab] OR "making decision*"[tiab] OR "making a decision"[tiab] OR "Decision support techniques"[Mesh] OR "Decision Support Systems, Clinical"[Mesh] OR "Decision aid*"[tiab] OR "decision process*"[tiab] OR "choice making"[tiab]))	130.261
**#3**	"Education"[Mesh:NoExp] OR “Curriculum”[Mesh] OR "Education, Medical, Continuing"[Mesh] OR "Education, Continuing"[Mesh:NoExp] OR "Patient Simulation"[Mesh] OR “patient simulation”[tiab] OR “continuing medical education”[tiab] OR “Lifelong learning” [tiab] OR “life long training” [tiab] OR “life-long education” [tiab] OR “life-long learning” [tiab] OR “lifelong education” [tiab] OR “lifelong training” [tiab] OR “tutorial*”[tiab] OR "role play*"[tiab] OR “workshop*”[tiab] OR “curricul*”[tiab] OR “course*”[tiab] OR “seminar*”[tiab] OR "e learning"[tiab] OR "elearning"[tiab] OR “professional retraining”[tiab] OR "decision aid*"[tiab] OR "discussion aid*"[tiab] OR "risk communication"[tiab] OR “continuing education”[tiab] OR<br>((educat*[tiab] OR “train*”[tiab] OR “teach*”[tiab] OR “learn*”[tiab] OR “didactic”[tiab]) AND (“skill*”[tiab] OR “simulat*”[tiab] OR “modul*”[tiab] OR “program*”[tiab] OR “intervention*”[tiab] OR “session*”[tiab]))	1.480.157
**#4**	"Clinical Trial" [Publication Type] OR "observational study"[publication type] OR "Randomized Controlled Trial" [Publication Type] OR "Qualitative Research"[Mesh] OR "Cross-Sectional Studies"[Mesh] OR "Focus Groups"[Mesh] OR "Surveys and Questionnaires"[Mesh] OR "Longitudinal Studies"[Mesh] OR "pre post"[tiab] OR "pre test"[tiab] OR "pre course"[tiab] OR "pre intervention" [tiab] OR "pre training"[tiab] OR "post course"[tiab] OR "post intervention"[tiab] OR "post training"[tiab] OR "post test"[tiab] OR "before after"[tiab] OR "before test"[tiab] OR "before course"[tiab] OR "before intervention"[tiab] OR "before training"[tiab] OR "after course"[tiab] OR "after intervention"[tiab] OR "after training"[tiab] OR "after test"[tiab] OR "RCT"[tiab] OR "RCTs"[tiab] OR "random*"[tiab] OR "controlled trial*"[tiab] OR "controlled stud*"[tiab] OR "cross sectional"[tiab] OR "observational stud*"[tiab] OR "qualitative stud*"[tiab] OR “Qualitative analys*”[tiab] OR “Qualitative Research” [tiab] OR "mixed method*"[tiab] OR "evaluation stud*"[tiab] OR "focus group*"[tiab] OR "program evaluation"[tiab] OR “Questionnaire*”[tiab] OR “survey*”[tiab] OR “interview*”[tiab] OR “longitudinal stud*”[tiab]	4.529.840
**#5**	"Review" [Publication Type] OR "Systematic Review" [Publication Type] OR "systematic review"[ti] OR "scoping review"[ti] OR "systematic literature review"[ti] OR "realist review"[ti]	3.208.989
**#6**	#1 AND #2 AND #3	7.683
**#7**	#6 AND #4	5.011
**#8**	#7 NOT #5	4.636

Embase

Concept 1: Primary care physicians
‘Physician’/de OR ‘General Practitioner’/exp OR ‘General Practice’/exp OR (‘general pract*’ OR ‘general doct*’ OR ‘family doct*’ OR ‘medical pract*’ OR ‘family pract*’ OR ‘physician*’ OR ‘health care provider*’ OR ‘healthcare provider*’ OR ‘healthcare professional*’ OR ‘health care professional*’ OR ‘primary care’):ti,ab,kw

Concept 2: SDM
(‘shared decision making’/exp OR ‘patient decision making’/exp OR ‘decision making’/de OR (‘Shared Decision*’ OR ‘sharing decision*’ OR ‘SDM’ OR ‘shared decisionmaking’ OR ‘patient decision-making’ OR ‘patient decisionmaking’ OR ‘participatory decision*’ OR ‘Patient-centered’ OR ‘Patient-centred’ OR ‘person-centered’ OR ‘person-centred’ OR ‘advanced communicat*’ OR ‘Participative Decision Making’ OR ‘informed decision-making’):ti,ab,kw) OR

((‘doctor patient relationship'/exp OR ‘Communication’/de OR 'patient participation'/exp OR (‘Physician Patient Relation*’ OR ‘Doctor patient relation*’ OR ‘clinician patient relation*’ OR ‘Communicat*’ OR ‘intercourse*’ OR ‘inter course*’ OR ‘interchang*’ OR ‘inter chang*’ OR ‘dialogu*’ OR ‘talk*’ OR ‘conversation*’ OR ‘participat*’ OR ‘involv*’ OR ‘patient focus*’ OR ‘engag*’):ti,ab,kw) AND ('shared decision making'/exp OR 'decision support system'/de OR (‘Decision making’ OR ‘decision aid’ OR ‘making decision*’ OR ‘making a decision’ OR ‘decision process*’ OR ‘choice making’):ti,ab,kw))

Concept 3 trainingsprogramma:

'continuing education'/de OR 'curriculum'/exp OR 'medical education'/de OR ‘education’/de OR 'education program'/exp OR 'lifelong learning'/exp OR 'patient simulation'/de OR (‘continuing medical education’ OR ‘Lifelong learning’ OR ‘life long training’ OR ‘life-long education’ OR ‘life-long learning’ OR ‘lifelong education’ OR ‘lifelong training’ OR tutorial* OR ‘role play*’ OR ‘workshop*’ OR curricul* OR course* OR seminar* OR ‘e learning’ OR ‘elearning’ OR ‘professional retraining’ OR ‘decision air/exp’ OR ‘decision aid*’ OR ‘discussion aid*’ OR ‘risk communication’ OR ‘continuing education’ OR ‘patient simulation’):ti,ab,kw OR ((educat* OR ‘train*’ OR teach* OR learn* OR didactic):ti,ab,kw AND (skill* OR simulat* OR modul* OR program* OR intervention*):ti,ab,kw)

Study design

'clinical trial'/de OR 'observational study'/exp OR 'randomized controlled trial'/exp OR 'qualitative research'/exp OR 'cross-sectional study'/exp OR 'questionnaire'/exp OR 'longitudinal study'/exp OR (‘pre post’ OR ‘pre test’ OR ‘pre course’ OR ‘pre intervention’ OR ‘pre training’ OR ‘post course’ OR ‘post intervention’ OR ‘post training’ OR ‘post test’ OR ‘before after’ OR ‘before test’ OR ‘before course’ OR ‘before intervention’ OR ‘before training’ OR ‘after course’ OR ‘after intervention’ OR ‘after training’ OR ‘after test’ OR ‘RCT’ OR ‘RCTs’ OR ‘random*’ OR ‘controlled trial*’ OR ‘controlled stud*’ OR ‘cross sectional’ OR ‘observ* stud*’ OR ‘Qualitative Research’ OR ‘qualitative stud*’ OR ‘Qualitative analys*’ OR ‘mixed method*’ OR ‘evaluation stud*’ OR ‘focus group*’ OR ‘program* evaluation*’ OR ‘Questionnaire*’ OR ‘survey*’ OR ‘interview*’ OR ‘longitudinal stud*’):ti,ab,kw

Exclude reviews

NOT ('conference abstract'/it OR 'review'/it OR 'systematic review'/it OR 'scoping review'/it OR 'realist review'/it)

Web of Science

Concept 1: Primary care physicians
TS=(“general pract*” OR “family doct*” OR “general doct*” OR “medical pract*” OR “family pract*” OR “physician*” OR “health care provider*” OR "healthcare provider*" OR "healthcare professional*" OR "health care professional*" OR “primary care”)

Concept 2: SDM
TS=(("Shared Decision*" OR "Sharing Decision*" OR "SDM" OR "patient decision-making" OR "patient decisionmaking" OR "participatory decision*"OR “Participative Decision Making” OR "Patient-centered" OR "Patient-centred" OR "person-centered" OR "person-centred" OR “advanced communicati*” OR “informed decision-making”) OR (("Physician-Patient Relation*" OR “Clinician-Patient Relation*” OR “Doctor-Patient Relation*” OR "Communicat*" OR "intercourse*" OR “inter course*” OR "interchang*" OR "inter chang*" OR "dialogu*" OR "talk*" OR "conversation*" OR "Patient Participation" OR "involv*" OR “patient-focus*” OR "engag*") AND ("Decision Making" OR "making decision*" OR "making a decision" OR "Decision support techniques" OR "Decision Support Systems, Clinical" OR "Decision aid*" OR "decision process*" OR "choice making")))

Concept 3 trainingsprogramma:

TS=((“Education” OR “Curriculum” OR "Patient Simulation" OR “continuing medical education” OR “Lifelong learning” OR “life long training” OR “life-long education” OR “life-long learning” OR “lifelong education” OR “lifelong training” OR tutorial* OR "role play*" OR “workshop*” OR curricul* OR course* OR seminar* OR "e learning" OR "elearning" OR “professional retraining” OR "decision aid*" OR "discussion aid*" OR "risk communication" OR “continuing education”) OR ((educat* OR “train*” OR teach* OR learn* OR didactic) AND (skill* OR simulat* OR modul* OR program* OR intervention*)))

Study design

TS=(("pre post" OR "pre test" OR "pre course" OR "pre intervention" OR "pre training" OR "post course" OR "post intervention" OR "post training" OR "post test" OR "before after" OR "before test" OR "before course" OR "before intervention" OR "before training" OR "after course" OR "after intervention" OR "after training" OR "after test" OR "RCT" OR "RCTs" OR "random*" OR "controlled trial*" OR "controlled stud*" OR "cross sectional" OR "observ* stud*" OR "qualitative stud*" OR “Qualitative analys*” OR “Qualitative Research” OR "mixed method*" OR "evaluation stud*" OR "focus group*" OR "program* evaluation*" OR Questionnaire* OR survey* OR interview* OR “longitudinal stud*”))

Exclude reviews

TI=("systematic review" OR "scoping review" OR "systematic literature review" OR "realist review") OR DT=("review")

Cochrane

Concept 1: Primary care physicians
(mh "Physicians") OR (mh "General Practitioners") OR (mh "Physicians, Family") OR (mh "General Practice") OR (mh "Physicians, Primary Care") OR ((general OR medical OR family) NEXT pract*):ti,ab,kw OR ((general OR family) NEXT doct*) OR “physician*” OR (("health care" OR healthcare) NEXT (provider* OR professional*) OR “primary care”):ti,ab,kw

Concept 2: SDM
((mh Decision Making, Shared”) OR (SDM OR Shared Decision OR Sharing Decision OR Participative Decision Making):ti,ab,kw OR (Patient NEXT (decision making OR decisionmaking OR centred* OR centered)):ti,ab,kw OR (person NEXT (centred OR centered)) OR ("participatory NEXT decision*"):ti,ab,kw OR (advanced NEXT communication) OR (“informed decision-making”):ti,ab,kw OR ((mh "Physician-Patient Relations") OR [mh ^"Communication"] OR [mh ^"Patient-centered care"] OR ((“physician OR clinician OR doctor) NEXT “patient NEXT relation*”) OR "Communicat*" OR "intercourse*" OR (inter NEXt course*) OR "interchang*"OR (inter NEXT chang*) OR "dialogu*"OR "talk*"OR "conversation*" OR "participat*" OR "involv*" OR "patient centered*"OR "patient centred" OR “patient-focus*”OR "engag*") AND ([mh ^"Decision Making"] OR "Decision making" OR "making decision*" OR "making a decision" OR (mh "Decision support techniques") OR (mh "Decision Support Systems, Clinical") OR "Decision aid*" OR "decision process*" OR "choice making")) AND ([mh ^"Education"] OR [mh “Curriculum”] OR [mh "Education, Medical, Continuing"] OR [mh "Education, Continuing"] OR (“continuing medical education” OR “Lifelong learning” OR “life long training” OR “life-long education” OR “life-long learning” OR “lifelong education” OR “lifelong training” OR tutorial* OR (Role NEXT play*) OR “workshop*” OR curricul* OR course* OR seminar* OR "e learning" OR "elearning" OR “professional retraining” OR (decision NEXT aid*) OR (discussion NEXT aid*) OR "risk communication" OR “continuing education”):ti,ab,kw OR ((educat* OR “train*” OR teach* OR learn* OR didactic) AND (skill* OR simulat* OR modul* OR program* OR intervention*)):ti,ab,kw) AND ((mh "Physicians") OR (mh "General Practitioners") OR (mh "Physicians, Family") OR (mh "General Practice") OR (mh "Physicians, Primary Care") OR ((general OR medical OR family) NEXT pract*):ti,ab,kw OR ((general OR medical OR family) NEXT doct*) OR (family NEXT (physician* OR doct*)) OR clinician* OR “physician*” OR “general physician” OR (("health care" OR healthcare) NEXT (provider* OR professional*) OR “primary care”):ti,ab,kw)

<u>Concept 3 training program:</u>

[mh ^"Education"] OR [mh “Curriculum”] OR [mh "Education, Medical, Continuing"] OR [mh "Education, Continuing"] OR (“continuing medical education” OR “Lifelong learning” OR “life long training” OR “life-long education” OR “life-long learning” OR “lifelong education” OR “lifelong training” OR tutorial* OR (Role NEXT play*) OR “workshop*” OR curricul* OR course* OR seminar* OR "e learning" OR "elearning" OR “professional retraining” OR (decision NEXT aid*) OR (discussion NEXT aid*) OR "risk communication" OR “continuing education”):ti,ab,kw OR ((educat* OR “train*” OR teach* OR learn* OR didactic) AND (skill* OR simulat* OR modul* OR program* OR intervention*)):ti,ab,kw

CINAHL

Concept 1: Primary care physicians

(MH “Physicians”) OR (MH "Primary Health Care") OR (MH "Physicians, Family") OR (MH "Family Practice") OR TI(“general pract*” OR “family doct*” OR “general doct*” OR “medical pract*” OR “family pract*” OR “physician*” OR “health care provider*” OR "healthcare provider*" OR "healthcare professional*" OR "health care professional*" OR “primary care”) OR AB(“general pract*” OR “physician*” OR “family doct*” OR “general doct*” OR “medical pract*” OR “family pract*” OR “health care provider*” OR "healthcare provider*" OR "healthcare professional*" OR "health care professional*" OR “primary care”)

Concept 2: SDM

(MH "Decision Making, Shared") OR TI("Shared Decision*" OR "Sharing Decision*" OR "SDM" OR "patient decision-making" OR "patient decisionmaking" OR "participatory decision*" OR “Participative Decision Making” OR "Patient-centered" OR "Patient-centred" OR "person-centered" OR "person-centred" OR “advanced communicati*” OR “informed decision-making”) OR AB("Shared Decision*" OR "Sharing Decision*" OR "SDM" OR "patient decision-making" OR "patient decisionmaking" OR "participatory decision*" OR "Patient-centered decision*" OR "Patient-centred decision*" OR "person-centered decision*" OR "person-centred decision*" OR “advanced communicati*”) OR ((MH "Physician-Patient Relations") OR (MH "Communication Skills") OR (MH "Patient Centered Care") OR TI(“Physician-Patient Relation*” OR “Clinician-Patient Relation*” OR “Doctor-Patient Relation*” OR "Communicat*" OR "intercourse*" OR “inter course*” OR "interchang*" OR "inter chang*" OR "dialogu*" OR "talk*" OR "conversation*" OR "participat*" OR "involv*" OR "patient centered*" OR "patient centred" OR “patient-focus*” OR "engag*") OR AB(“Physician-Patient Relation*” OR “Clinician-Patient Relation*” OR “Doctor-Patient Relation*” OR "Communicat*" OR "intercourse*" OR “inter course*” OR "interchang*" OR "inter chang*" OR "dialogu*" OR "talk*" OR "conversation*" OR "participat*" OR "involv*" OR "patient centered*" OR "patient centred" OR “patient-focus*” OR "engag*") AND ((MH "Decision Making”) OR (MH "Decision Support Techniques") OR (MH "Decision Support Systems, Clinical") OR TI("Decision making" OR "making decision*" OR "making a decision" OR "Decision aid*" OR "decision process*" OR "choice making") OR AB("Decision making" OR "making decision*" OR "making a decision" OR "Decision aid*" OR "decision process*" OR "choice making")))

Concept 3 training program:

(MH "Education") OR (MH "Course Evaluation") OR (MH "Curriculum") OR (MH "Education, Medical, Continuing") OR (MH "Education, Medical") OR (MH "Patient Simulation") OR TI(tutorial* OR "role play*" OR “workshop*” OR curricul* OR course* OR seminar* OR "e learning" OR "elearning" OR "decision aid*" OR "discussion aid*" OR "risk communication" OR “continuing medical education” OR “Lifelong learning” OR “life long training” OR “life-long education” OR “life-long learning” OR “lifelong education” OR “lifelong training” OR “continuing education”) OR AB(tutorial* OR "role play*" OR “workshop*” OR curricul* OR course* OR seminar* OR "e learning" OR "elearning" OR “professional retraining” OR "decision aid*" OR "discussion aid*" OR "risk communication" OR “continuing medical education” OR “Lifelong learning” OR “life long training” OR “life-long education” OR “life-long learning” OR “lifelong education” OR “lifelong training” OR “continuing education”) OR ((TI(educat* OR “train*” OR teach* OR learn* OR didactic) OR AB(educat* OR “train*” OR teach* OR learn* OR didactic)) AND (TI(skill* OR simulat* OR modul* OR program* OR intervention*) OR AB(skill* OR simulat* OR modul* OR program* OR intervention*)))

Study design

PT ("Clinical Trial" OR "Randomized Controlled Trial") OR TI ("pre post" OR "pre test" OR "pre course" OR "pre intervention" OR "pre training" OR "post course" OR "post intervention" OR "post training" OR "post test" OR "before after" OR "before test" OR "before course" OR "before intervention" OR "before training" OR "after course" OR "after intervention" OR "after training" OR "after test" OR "RCT" OR "RCTs" OR "random*" OR "controlled trial*" OR "controlled stud*" OR "cross sectional" OR "observ* stud*" OR "qualitative stud*" OR “Qualitative analys*” OR “Qualitative Research” OR "mixed method*" OR "evaluation stud*" OR "focus group*" OR "program* evaluation*" OR Questionnaire* OR survey* OR interview* OR “longitudinal stud*”) OR AB("pre post" OR "pre test" OR "pre course" OR "pre intervention" OR "pre training" OR "post course" OR "post intervention" OR "post training" OR "post test" OR "before after" OR "before test" OR "before course" OR "before intervention" OR "before training" OR "after course" OR "after intervention" OR "after training" OR "after test" OR "RCT" OR "RCTs" OR "random*" OR "controlled trial*" OR "controlled stud*" OR "cross sectional" OR "observ* stud*" OR "qualitative stud*" OR “Qualitative analys*” OR “Qualitative Research” OR "mixed method*" OR "evaluation stud*" OR "focus group*" OR "program* evaluation*" OR Questionnaire* OR survey* OR interview* OR “longitudinal stud*”)

Exclude reviews

PT (“Review” OR “Systematic Review”) OR TI("systematic review" OR "scoping review" OR "systematic literature review" OR "realist review")

Scopus

Concept 1: Primary care physicians
TITLE-ABS(“general pract*” OR “family doctor*” OR “medical pract*” OR “family pract*” OR “physician*” OR “health care provider*” OR “healthcare provider*” OR “healthcare professional*” OR “health care professional*” OR “primary care” OR “general doct*”) OR AUTHKEY(“general pract*” OR “family doctor*” OR “medical pract*” OR “family pract*” OR “physician*” OR “health care provider*” OR “healthcare provider*” OR “healthcare professional*” OR “health care professional*” OR “primary care” OR “general doct*”)

Concept 2: SDM
TITLE-ABS("Shared Decision*" OR "Sharing Decision*" OR “Participative Decision Making” OR "SDM" OR "patient decision-making" OR "patient decisionmaking" OR "participatory decision*" OR "Patient-centered" OR "Patient-centred" OR "person-centered" OR "person-centred" OR “advanced communicati*” OR “informed decision-making”) OR (("Physician-Patient Relation*” OR “Clinician-Patient Relation*” OR “Doctor-Patient Relation*” OR "Communicat*" OR "intercourse*" OR “inter course*” OR "interchang*" OR "inter chang*" OR "dialogu*" OR "talk*" OR "conversation*" OR "participat*" OR "involv*" OR "patient centered*" OR "patient centred" OR “patient-focus*” OR "engag*") AND ("Decision making" OR "making decision*" OR "making a decision" OR "Decision Support techniques OR "Decision aid*" OR "decision process*" OR "choice making")) OR AUTHKEY("Shared Decision*" OR "Sharing Decision*" OR “Participative Decision Making” OR "SDM" OR "patient decision-making" OR "patient decisionmaking" OR "participatory decision*" OR "Patient-centered" OR "Patient-centred" OR "person-centered" OR "person-centred" OR “advanced communicati*” OR “informed decision-making”) OR (("Physician-Patient Relation*" OR “Clinician-Patient Relation*” OR “Doctor-Patient Relation*” OR "Communicat*" OR "intercourse*" OR “inter course*” OR "interchang*" OR "inter chang*" OR "dialogu*" OR "talk*" OR "conversation*" OR "participat*" OR "involv*" OR "patient centered*" OR "patient centred" OR “patient-focus*” OR "engag*") AND ("Decision making" OR "making decision*" OR "making a decision" OR "Decision Support techniques OR "Decision aid*" OR "decision process*" OR "choice making"))

<u>Concept 3 trainingprogram:</u>

TITLE-ABS("Education" OR “Curriculum” OR "Patient Simulation" OR “continuing medical education” OR “Lifelong learning” OR “life long training” OR “life-long education” OR “life-long learning” OR “lifelong education” OR “lifelong training” OR tutorial* OR "role play*" OR “workshop*” OR curricul* OR course* OR seminar* OR "e learning" OR "elearning" OR “professional retraining” OR "decision aid*" OR "discussion aid*" OR "risk communication" OR “continuing education” OR ((educat* OR “train*” OR teach* OR learn* OR didactic) AND (skill* OR simulat* OR modul* OR program* OR intervention*))) OR AUTHKEY("Education" OR “Curriculum” OR "Patient Simulation" OR “continuing medical education” OR “Lifelong learning” OR “life long training” OR “life-long education” OR “life-long learning” OR “lifelong education” OR “lifelong training” OR tutorial* OR "role play*" OR “workshop*” OR curricul* OR course* OR seminar* OR "e learning" OR "elearning" OR “professional retraining” OR "decision aid*" OR "discussion aid*" OR "risk communication" OR ((educat* OR “train*” OR teach* OR learn* OR didactic) AND (skill* OR simulat* OR modul* OR program* OR intervention*)))

<u>Study design</u>

TITLE-ABS(“pre post" OR "pre test" OR "pre course" OR "pre intervention" OR "pre training" OR "post course" OR "post intervention" OR "post training" OR "post test" OR "before after" OR "before test" OR "before course" OR "before intervention" OR "before training" OR "after course" OR "after intervention" OR "after training" OR "after test" OR "RCT" OR "RCTs" OR "random*" OR "controlled trial*" OR "controlled stud*" OR "cross sectional" OR "observ* stud*" OR "qualitative stud*" OR “Qualitative analys*” OR “Qualitative Research” OR "mixed method*" OR "evaluation stud*" OR "focus group*" OR "program* evaluation*" OR Questionnaire* OR survey* OR interview* OR “longitudinal stud*”) OR AUTHKEY (“pre post" OR "pre test" OR "pre course" OR "pre intervention" OR "pre training" OR "post course" OR "post intervention" OR "post training" OR "post test" OR "before after" OR "before test" OR "before course" OR "before intervention" OR "before training" OR "after course" OR "after intervention" OR "after training" OR "after test" OR "RCT" OR "RCTs" OR "random*" OR "controlled trial*" OR "controlled stud*" OR "cross sectional" OR "observ* stud*" OR "qualitative stud*" OR “Qualitative analys*” OR “Qualitative Research” OR "mixed method*" OR "evaluation stud*" OR "focus group*" OR "program* evaluation*" OR Questionnaire* OR survey* OR interview* OR “longitudinal stud*”)

Exclude reviews AND NOT

DOCTYPE (re) OR TITLE(“Systematic Review” OR “systematic review” OR “scoping review” OR “systematic literature review” OR “realist review”)

ERIC (via OVID)

Concept 1: Primary care physicians
exp Physicians/ or exp "Family Practice (Medicine)"/ or general practitioner.mp. or exp Primary Health Care/ OR (General practi* OR physician* OR family doct* OR general doct* OR medical pract* OR family pract* OR health care provider* OR healthcare provider* OR healthcare professional* OR health care professional* OR primary care).ti,ab,id.

Concept 2: SDM
exp Participative Decision Making/ or exp Decision Making/ OR (Shared Decision* OR Sharing Decision* OR SDM OR patient decision-making OR patient decisionmaking OR participatory decision* OR Patient-centered OR Patient-centred OR person-centered OR person-centred OR advanced communicati* OR informed decision-making).mp. OR ((physician patient relationship/ OR (Physician-Patient Relation* OR Clinician-Patient Relation* OR Doctor-Patient Relation* OR communication OR Communicat* OR intercourse* OR inter course* OR interchang* OR inter chang* OR dialogu* OR talk* OR conversation* OR participat* OR involv* OR patient centered* OR patient centred OR patient-focus* OR engag*).ti,ab,id.) AND (exp Participative Decision Making/ or exp Decision Making/ OR (Decision making OR making decision* OR making a decision Decision aid* OR decision process* OR choice making).ti,ab,id.))

Concept 3 training program:

exp Medical Education/ or exp Professional Continuing Education/ or exp Continuing Education/ or curriculum/ or courses/ or curriculum evaluation/ or education/ OR (continuing medical education OR Lifelong learning OR life long training OR life-long education OR life-long learning OR lifelong education OR lifelong training OR tutorial* OR role play* OR workshop* OR curricul* OR course* OR seminar* OR e learning OR elearning OR professional retraining OR decision aid* OR discussion aid* OR risk communication OR continuing education).mp. OR ((educat* OR train* OR teach* OR learn* OR didactic) AND (skill* OR simulat* OR modul* OR program* OR intervention*).ti,ab,id.)

Study design

(pre post OR pre test OR pre course OR pre intervention OR pre training OR post course OR post intervention OR post training OR post test OR before after OR before test OR before course OR before intervention OR before training OR after course OR after intervention OR after training OR after test OR RCT OR RCTs OR random* OR controlled trial* OR controlled stud* OR cross sectional OR observ* stud* OR qualitative stud* OR Qualitative analys* OR Qualitative Research OR mixed method* OR evaluation stud* OR focus group* OR program* evaluation* OR Questionnaire* OR survey* OR interview* OR longitudinal stud*).ti,ab,id.
