# Supplementary material for: A systematic review of shared decision making training programs for general practitioners
Source: BMC Med Educ. 2024 May 29;24:592. doi: 10.1186/s12909-024-05557-1 (PMC11137915; doi:10.1186/s12909-024-05557-1)
Supplement: Supplementary file 2 — Supplementary Material 2. [file 12909_2024_5557_MOESM2_ESM.pdf]

Additional file 2: Forest plots of all studies categorized by Kirkpatrick level

Kirkpatrick level 2

Clinicians' intention to engage in SDM

	Experimental			Control				Mean Difference
Study or Subgroup	Mean	SD	Total	Mean	SD	Total	Weight	IV, Fixed, 95% CI
Légaré 2012	1.7	0.9	160	1.8	0.7	108	100.0%	-0.10 [-0.29, 0.09]
**Total (95% CI)**			**160**			**108**	**100.0%**	**-0.10 [-0.29, 0.09]**

Heterogeneity: Not applicable
Test for overall effect: Z = 1.02 (P = 0.31)

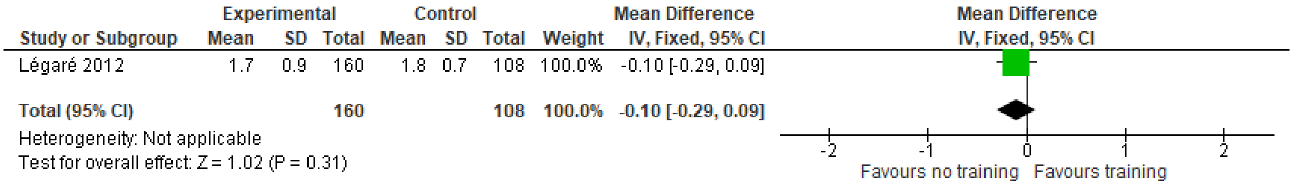

Figure A2-1: Clinicians' intention to engage in SDM.

Kirkpatrick level 4

Patient satisfaction with consultation

	Training			No training				Std. Mean Difference
Study or Subgroup	Mean	SD	Total	Mean	SD	Total	Weight	IV, Random, 95% CI
Loh 2007	29.8	2.7	191	27	3.6	96	26.5%	0.92 [0.67, 1.18]
Roter 2012	4.5	0.47	97	4.2	0.16	97	26.0%	0.85 [0.56, 1.15]
Stewart 2007	82.06	5.8	11	77.78	8.07	58	20.2%	0.54 [-0.11, 1.20]
Wilkes 2013	18	2	188	18	3	291	27.3%	0.00 [-0.18, 0.18]
**Total (95% CI)**			**487**			**542**	**100.0%**	**0.58 [0.03, 1.12]**

Heterogeneity: Tau² = 0.28; Chi² = 43.21, df = 3 (P < 0.00001); I² = 93%
Test for overall effect: Z = 2.05 (P = 0.04)

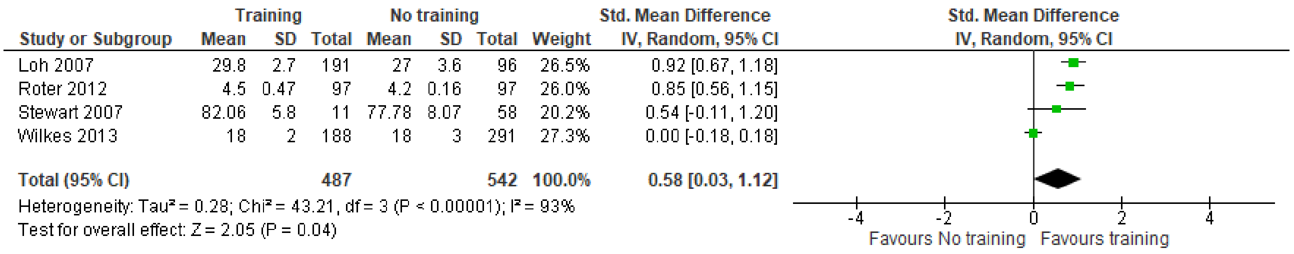

Figure A2-2: Patient satisfaction with consultation.

Patient satisfaction with consultation (categorical)

	Training		No training			Risk Difference
Study or Subgroup	Events	Total	Events	Total	Weight	M-H, Random, 95% CI
Cals 2007	144	201	151	230	32.1%	0.06 [-0.03, 0.15]
Kunneman 2022	146	153	124	126	35.9%	-0.03 [-0.07, 0.01]
Sepucha 2022	157	232	124	222	32.0%	0.12 [0.03, 0.21]
**Total (95% CI)**		**586**		**578**	**100.0%**	**0.05 [-0.09, 0.18]**
Total events	447		399			

Heterogeneity: Tau² = 0.01; Chi² = 21.89, df = 2 (P < 0.0001); I² = 91%
Test for overall effect: Z = 0.67 (P = 0.50)

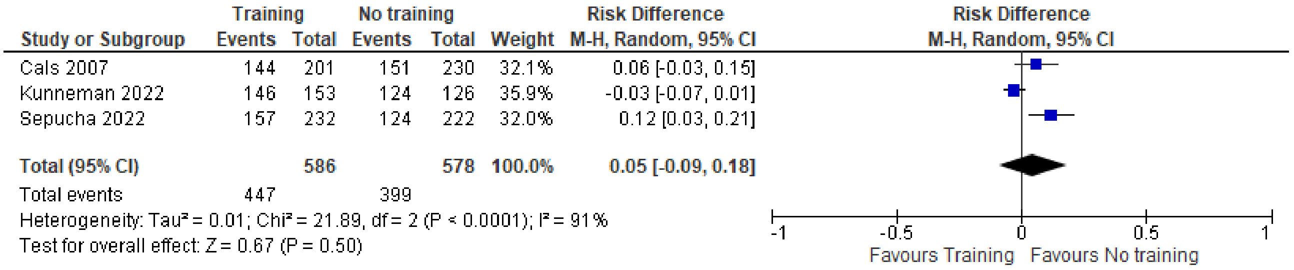

Figure A2-3: Patient satisfaction with consultation (Categorical outcome)

Physician satisfaction with consultation

	training		No training			Risk Difference
Study or Subgroup	Events	Total	Events	Total	Weight	M-H, Random, 95% CI
Kunneman 2020	400	453	277	448	50.5%	0.26 [0.21, 0.32]
Kunneman 2022	155	176	133	155	49.5%	0.02 [-0.05, 0.10]
**Total (95% CI)**		**629**		**603**	**100.0%**	**0.14 [-0.10, 0.39]**
Total events	555		410			

Heterogeneity: Tau² = 0.03; Chi² = 28.29, df = 1 (P < 0.00001); I² = 96%
Test for overall effect: Z = 1.18 (P = 0.24)

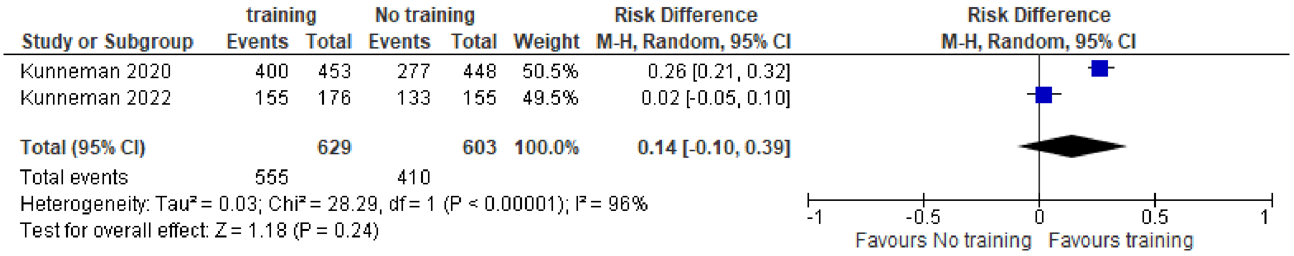

Figure A2-4: Physician satisfaction with consultation (Categorical outcome)

Decisional regret

Study or Subgroup	Training Mean	Training SD	Training Total	No training Mean	No training SD	No training Total	Weight	Std. Mean Difference IV, Random, 95% CI
Hirsch 2008	14.69	372	385	18.08	372	377	54.0%	-0.01 [-0.15, 0.13]
Légaré 2012	12.4	19.1	163	7.6	13.7	165	46.0%	0.29 [0.07, 0.51]
**Total (95% CI)**			**548**			**542**	**100.0%**	**0.13 [-0.16, 0.42]**

Heterogeneity: Tau² = 0.04; Chi² = 5.04, df = 1 (P = 0.02); I² = 80%
Test for overall effect: Z = 0.86 (P = 0.39)

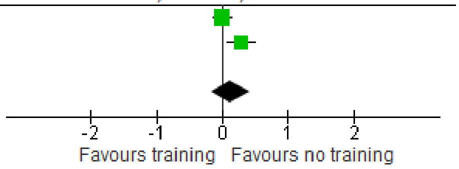

Figure A2-5: Decisional regret

Patients' intention to engage in SDM

Study or Subgroup	Experimental Mean	Experimental SD	Experimental Total	Control Mean	Control SD	Control Total	Weight	Mean Difference IV, Fixed, 95% CI
Légaré 2012	2.1	1.1	163	1.9	1.2	165	100.0%	0.20 [-0.05, 0.45]
**Total (95% CI)**			**163**			**165**	**100.0%**	**0.20 [-0.05, 0.45]**

Heterogeneity: Not applicable
Test for overall effect: Z = 1.57 (P = 0.12)

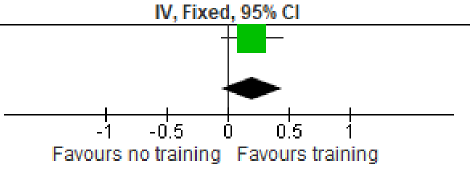

Figure A2-6: Patients' intention to engage in SDM.

Quality of life

Study or Subgroup	Training Mean	Training SD	Training Total	No training Mean	No training SD	No training Total	Weight	Std. Mean Difference IV, Random, 95% CI
**13.1.1 QOL Mental**								
Cooper 2011	51.6	10.3	83	50	11.5	55	13.5%	0.15 [-0.19, 0.49]
Elwyn 2004	50.4	10.9	171	47.8	11.2	124	26.5%	0.24 [0.00, 0.47]
Légaré 2012	50.8	9.3	181	51.2	8.4	178	31.9%	-0.05 [-0.25, 0.16]
Loh 2007	48.9	34.2	191	50.6	35.3	96	24.1%	-0.05 [-0.29, 0.20]
Stewart 2007	0.4	0.4	11	0.46	0.38	58	4.0%	-0.15 [-0.80, 0.49]
**Subtotal (95% CI)**			**637**			**511**	**100.0%**	**0.05 [-0.08, 0.18]**
Heterogeneity: Tau² = 0.00; Chi² = 4.59, df = 4 (P = 0.33); I² = 13%								
Test for overall effect: Z = 0.74 (P = 0.46)								
**13.1.2 QOL Physical**								
Cooper 2011	39.8	12.5	83	40.4	11.4	55	17.0%	-0.05 [-0.39, 0.29]
Elwyn 2004	42.5	11.8	171	41.9	13.1	124	37.0%	0.05 [-0.18, 0.28]
Légaré 2012	49.4	7.5	181	48.2	7.8	178	46.0%	0.16 [-0.05, 0.36]
**Subtotal (95% CI)**			**435**			**357**	**100.0%**	**0.08 [-0.06, 0.22]**
Heterogeneity: Tau² = 0.00; Chi² = 1.15, df = 2 (P = 0.56); I² = 0%								
Test for overall effect: Z = 1.14 (P = 0.26)								
**13.1.3 QOL - Total**								
Tilburgs 2020	58.1	6.9	73	57.8	7.1	67	100.0%	0.04 [-0.29, 0.37]
**Subtotal (95% CI)**			**73**			**67**	**100.0%**	**0.04 [-0.29, 0.37]**
Heterogeneity: Not applicable								
Test for overall effect: Z = 0.25 (P = 0.80)								

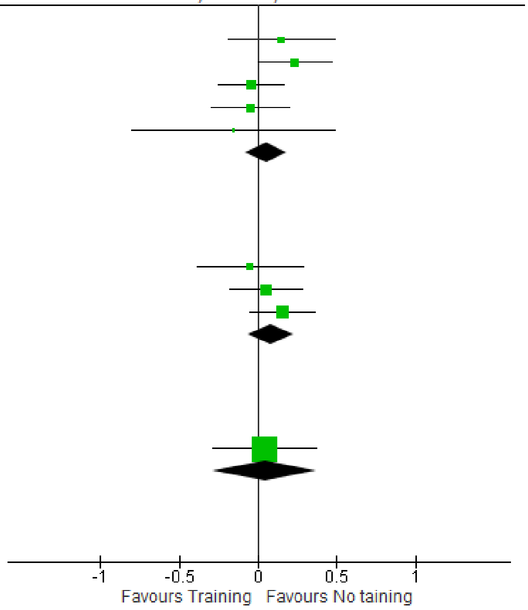

Test for subgroup differences: Chi² = 0.12, df = 2 (P = 0.94), I² = 0%

Figure A2-7: Quality of life.
