# Supplementary material for: A systematic review of shared decision making training programs for general practitioners
Source: BMC Med Educ. 2024 May 29;24:592. doi: 10.1186/s12909-024-05557-1 (PMC11137915; doi:10.1186/s12909-024-05557-1)
Supplement: Supplementary file 3 — Supplementary Material 3. [file 12909_2024_5557_MOESM3_ESM.pdf]

Additional file 3: forest plots of all studies: online learning

Patient reported outcome measure

Study or Subgroup	SDM Training Events	SDM Training Total	No SDM training Events	No SDM training Total	Weight	Risk Difference M-H, Random, 95% CI	Year
Mathers 2012	25	92	28	77	51.8%	-0.09 [-0.23, 0.05]	2012
Cooper 2013	49	67	41	65	48.2%	0.10 [-0.06, 0.26]	2013
**Total (95% CI)**		**159**		**142**	**100.0%**	**0.00 [-0.19, 0.19]**	
Total events	74		69				

Heterogeneity: Tau² = 0.01; Chi² = 3.17, df = 1 (P = 0.07); I² = 68%
Test for overall effect: Z = 0.01 (P = 0.99)

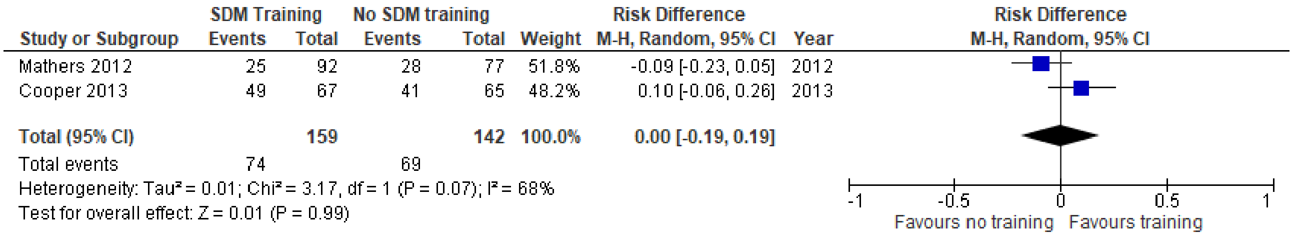

Figure A3-1: Shared decision making – patient reported outcomes (categorical outcomes).

Healthcare professional reported outcome measure

Study or Subgroup	SDM Training Mean	SDM Training SD	SDM Training Total	No SDM training Mean	No SDM training SD	No SDM training Total	Weight	Mean Difference IV, Random, 95% CI	Year
Wilkes 2013	15.9	1.3	41	16.1	1.6	43	100.0%	-0.20 [-0.82, 0.42]	2013
**Total (95% CI)**			**41**			**43**	**100.0%**	**-0.20 [-0.82, 0.42]**	

Heterogeneity: Not applicable
Test for overall effect: Z = 0.63 (P = 0.53)

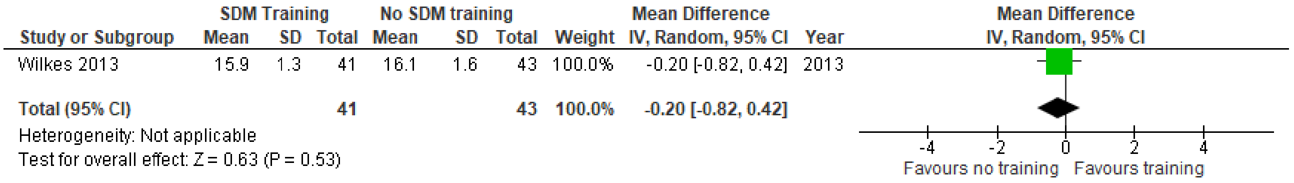

Figure A3-2: Shared decision making – Healthcare professional reported outcomes

Patient satisfaction with consultation

Study or Subgroup	SDM Training Mean	SDM Training SD	SDM Training Total	No SDM training Mean	No SDM training SD	No SDM training Total	Weight	Std. Mean Difference IV, Random, 95% CI	Year
Roter 2012	4.5	0.47	97	4.2	0.16	97	49.0%	0.85 [0.56, 1.15]	2012
Wilkes 2013	18	2	188	18	3	291	51.0%	0.00 [-0.18, 0.18]	2013
**Total (95% CI)**			**285**			**388**	**100.0%**	**0.42 [-0.42, 1.25]**	

Heterogeneity: Tau² = 0.35; Chi² = 23.16, df = 1 (P < 0.00001); I² = 96%
Test for overall effect: Z = 0.98 (P = 0.33)

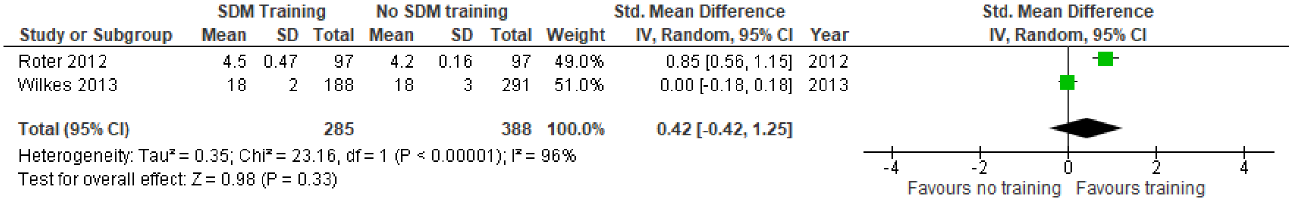

Figure A3-3: Patient satisfaction with consultation.

Study or Subgroup	SDM Training Events	SDM Training Total	No SDM training Events	No SDM training Total	Weight	Risk Difference M-H, Fixed, 95% CI
Sepucha 2022	157	232	124	222	100.0%	0.12 [0.03, 0.21]
**Total (95% CI)**		**232**		**222**	**100.0%**	**0.12 [0.03, 0.21]**
Total events	157		124			

Heterogeneity: Not applicable
Test for overall effect: Z = 2.61 (P = 0.009)

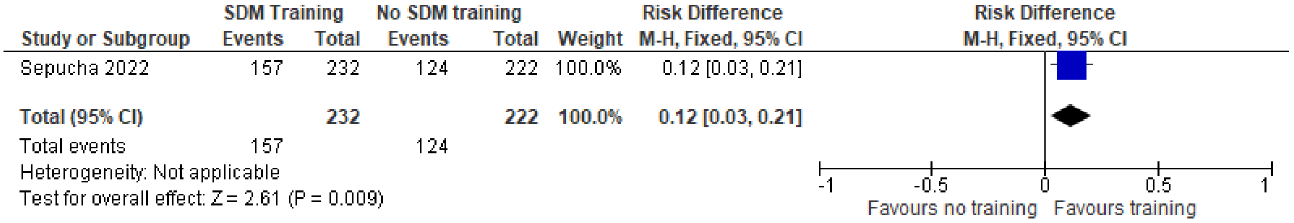

Figure A3-4: Patient satisfaction with consultation (categorical outcome).

Physician satisfaction with consultation

Study or Subgroup	SDM Training Events	SDM Training Total	No SDM training Events	No SDM training Total	Weight	Risk Difference M-H, Fixed, 95% CI
Kunneman 2020	400	453	277	448	100.0%	0.26 [0.21, 0.32]
**Total (95% CI)**		**453**		**448**	**100.0%**	**0.26 [0.21, 0.32]**
Total events	400		277			

Heterogeneity: Not applicable
Test for overall effect: Z = 9.63 (P < 0.00001)

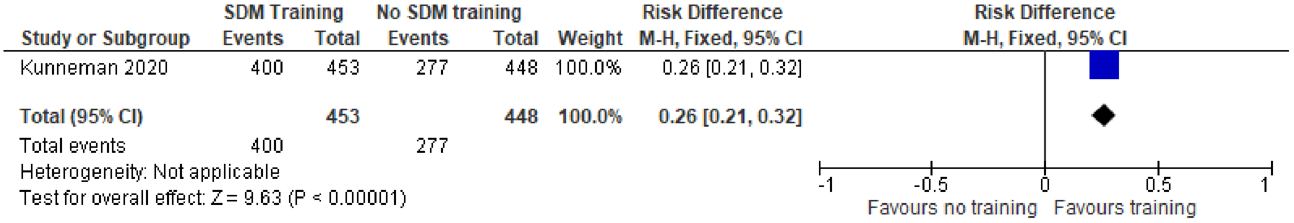

Figure A3-5: Physician satisfaction with consultation
