# Supplementary material for: A systematic review of shared decision making training programs for general practitioners
Source: BMC Med Educ. 2024 May 29;24:592. doi: 10.1186/s12909-024-05557-1 (PMC11137915; doi:10.1186/s12909-024-05557-1)
Supplement: Supplementary file 4 — Supplementary Material 4. [file 12909_2024_5557_MOESM4_ESM.pdf]

Additional file 4: forest plots of all studies: live learning

Patient reported outcome measure

Study or Subgroup	Experimental Events	Experimental Total	Control Events	Control Total	Weight	Risk Difference M-H, Fixed, 95% CI
Tai-Seale 2016	11	14	11	17	100.0%	0.14 [-0.17, 0.45]
**Total (95% CI)**		**14**		**17**	**100.0%**	**0.14 [-0.17, 0.45]**
Total events	11		11			

Heterogeneity: Not applicable
Test for overall effect: Z = 0.87 (P = 0.38)

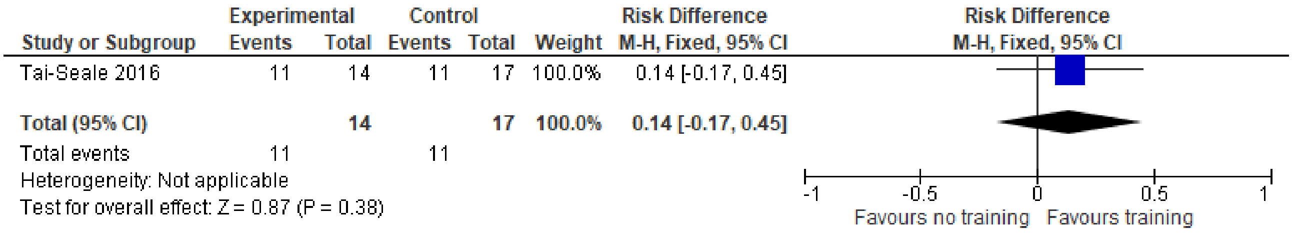

Figure A4-1: Shared decision making – patient reported outcome (categorical)

Healthcare professional reported outcome measure

Study or Subgroup	Experimental Mean	Experimental SD	Experimental Total	Control Mean	Control SD	Control Total	Weight	Std. Mean Difference IV, Random, 95% CI	Year
Hirsch 2008	1.66	0.45	44	1.65	0.48	47	80.7%	0.02 [-0.39, 0.43]	2008
Ngu 2022	78.1	14.1	11	73.2	19.8	11	19.3%	0.27 [-0.57, 1.11]	2022
**Total (95% CI)**			**55**			**58**	**100.0%**	**0.07 [-0.30, 0.44]**	

Heterogeneity: Tau² = 0.00; Chi² = 0.28, df = 1 (P = 0.60); I² = 0%
Test for overall effect: Z = 0.37 (P = 0.71)

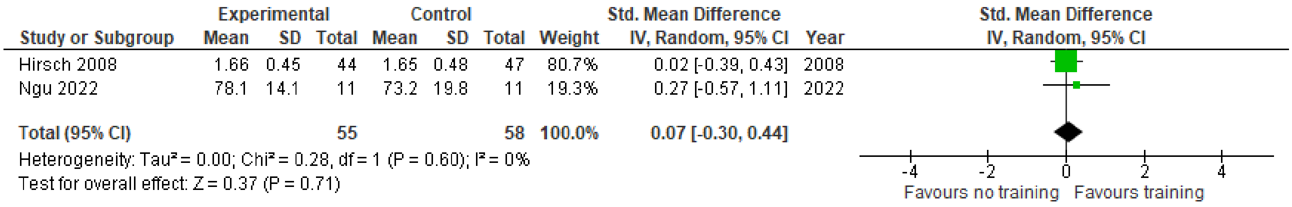

Figure A4-2: Shared decision making - healthcare professional reported outcome

Patient satisfaction with consultation

Study or Subgroup	Experimental Events	Experimental Total	Control Events	Control Total	Weight	Risk Difference M-H, Random, 95% CI
Cals 2007	144	201	151	230	45.8%	0.06 [-0.03, 0.15]
Kunneman 2022	146	153	124	126	54.2%	-0.03 [-0.07, 0.01]
**Total (95% CI)**		**354**		**356**	**100.0%**	**0.01 [-0.12, 0.15]**
Total events	290		275			

Heterogeneity: Tau² = 0.01; Chi² = 7.90, df = 1 (P = 0.005); I² = 87%
Test for overall effect: Z = 0.16 (P = 0.87)

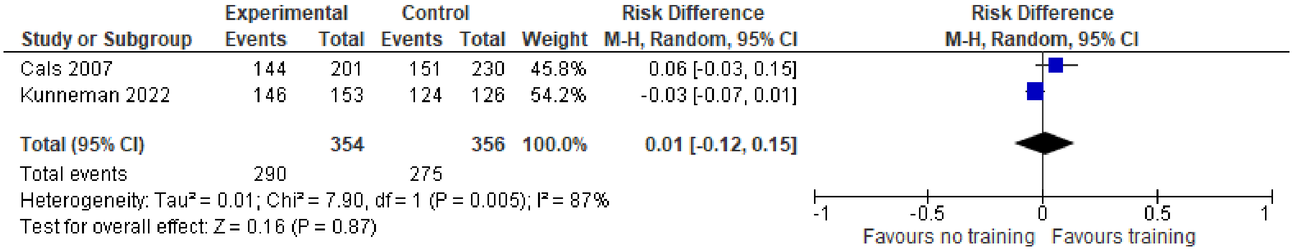

Figure A4-3: Patient satisfaction with consultation (categorical)

Decisional regret

Study or Subgroup	Experimental Mean	Experimental SD	Experimental Total	Control Mean	Control SD	Control Total	Weight	Mean Difference IV, Fixed, 95% CI
Hirsch 2008	14.69	372	385	18.08	372	377	100.0%	-3.39 [-56.22, 49.44]
**Total (95% CI)**			**385**			**377**	**100.0%**	**-3.39 [-56.22, 49.44]**

Heterogeneity: Not applicable
Test for overall effect: Z = 0.13 (P = 0.90)

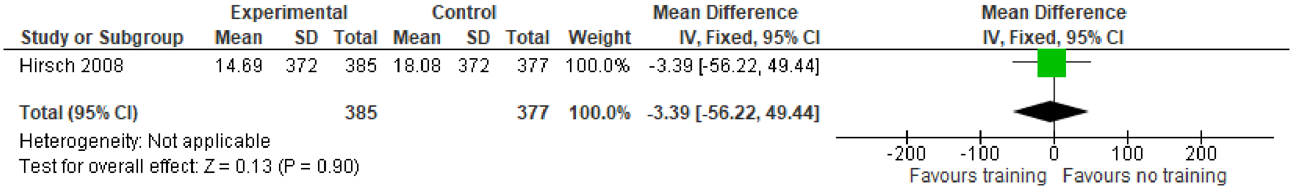

Figure A4-4: Decisional regret

Decisional conflict

Study or Subgroup	Experimental Mean	Experimental SD	Experimental Total	Control Mean	Control SD	Control Total	Weight	Mean Difference IV, Random, 95% CI	Year
Adarkwah 2016	15.4	14	147	14.8	12.4	157	100.0%	0.60 [-2.38, 3.58]	2016
**Total (95% CI)**			**147**			**157**	**100.0%**	**0.60 [-2.38, 3.58]**	

Heterogeneity: Not applicable
Test for overall effect: Z = 0.39 (P = 0.69)

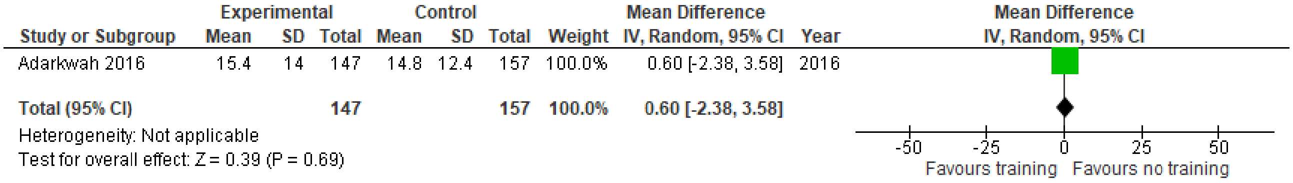

Figure A4-5: Decisional conflict

Clinician satisfaction with consultation

Study or Subgroup	Experimental Events	Experimental Total	Control Events	Control Total	Weight	Risk Difference M-H, Fixed, 95% CI
Kunneman 2022	155	176	133	155	100.0%	0.02 [-0.05, 0.10]
**Total (95% CI)**		**176**		**155**	**100.0%**	**0.02 [-0.05, 0.10]**
Total events	155		133			
Heterogeneity: Not applicable						
Test for overall effect: Z = 0.61 (P = 0.54)						

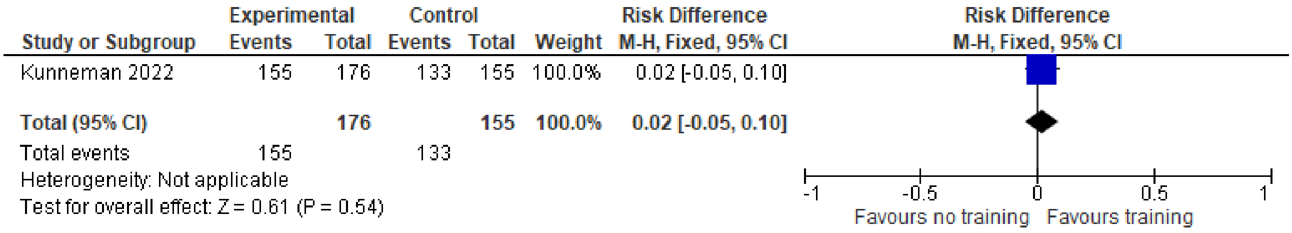

Figure A4-6: Clinician satisfaction with consultation

Quality of life

Study or Subgroup	Experimental Mean	Experimental SD	Experimental Total	Control Mean	Control SD	Control Total	Weight	Std. Mean Difference IV, Random, 95% CI
**3.10.1 Mental**								
Elwyn 2004	50.4	10.9	171	47.8	11.2	124	27.3%	0.24 [0.00, 0.47]
Loh 2007	45.9	34.2	191	50.6	35.3	96	25.1%	-0.14 [-0.38, 0.11]
Stewart 2007	0.4	0.4	11	0.46	0.38	58	4.7%	-0.15 [-0.80, 0.49]
**Subtotal (95% CI)**			**373**			**278**	**57.0%**	**0.02 [-0.27, 0.31]**
Heterogeneity: Tau² = 0.04; Chi² = 5.03, df = 2 (P = 0.08); I² = 60%								
Test for overall effect: Z = 0.14 (P = 0.89)								
**3.10.2 Physical**								
Elwyn 2004	42.5	11.8	171	41.9	13.1	124	27.4%	0.05 [-0.18, 0.28]
**Subtotal (95% CI)**			**171**			**124**	**27.4%**	**0.05 [-0.18, 0.28]**
Heterogeneity: Not applicable								
Test for overall effect: Z = 0.41 (P = 0.68)								
**3.10.3 Quality of life**								
Tilburgs 2020	58.1	6.9	73	57.8	7.1	67	15.6%	0.04 [-0.29, 0.37]
**Subtotal (95% CI)**			**73**			**67**	**15.6%**	**0.04 [-0.29, 0.37]**
Heterogeneity: Not applicable								
Test for overall effect: Z = 0.25 (P = 0.80)								
**Total (95% CI)**			**617**			**469**	**100.0%**	**0.04 [-0.10, 0.19]**
Heterogeneity: Tau² = 0.01; Chi² = 5.03, df = 4 (P = 0.28); I² = 20%								
Test for overall effect: Z = 0.59 (P = 0.56)								
Test for subgroup differences: Chi² = 0.02, df = 2 (P = 0.99), I² = 0%								

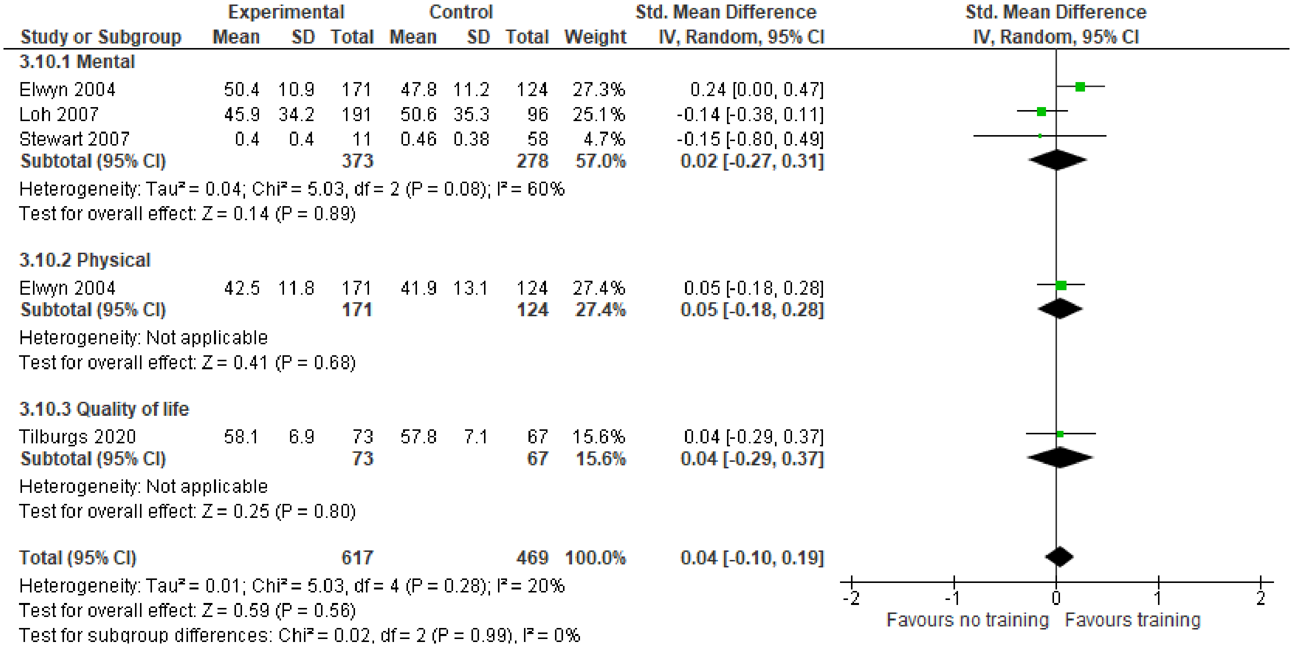

Figure A4-7: Quality of life.
