# Supplementary material for: A systematic review of shared decision making training programs for general practitioners
Source: BMC Med Educ. 2024 May 29;24:592. doi: 10.1186/s12909-024-05557-1 (PMC11137915; doi:10.1186/s12909-024-05557-1)
Supplement: Supplementary file 5 — Supplementary Material 5. [file 12909_2024_5557_MOESM5_ESM.pdf]

Additional file 5: forest plots of all studies: blended learning

Clinicians' intention to engage in SDM

Study or Subgroup	Experimental Mean	Experimental SD	Experimental Total	Control Mean	Control SD	Control Total	Weight	Mean Difference IV, Fixed, 95% CI
Légaré 2012	1.7	0.9	160	1.8	0.7	108	100.0%	-0.10 [-0.29, 0.09]
**Total (95% CI)**			**160**			**108**	**100.0%**	**-0.10 [-0.29, 0.09]**

Heterogeneity: Not applicable
Test for overall effect: Z = 1.02 (P = 0.31)

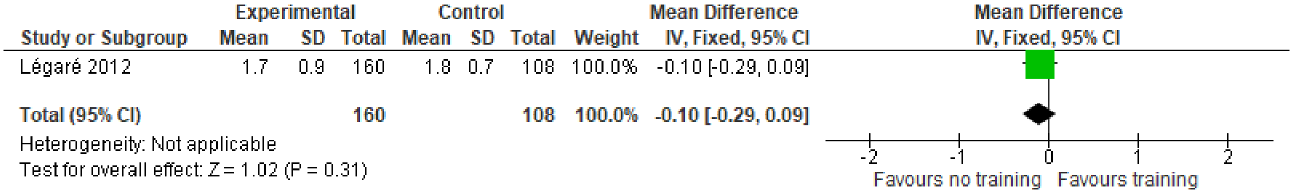

Figure A5-1: Clinicians' intention to engage in SDM.

Patient reported outcome measure

Study or Subgroup	Experimental Events	Experimental Total	Control Events	Control Total	Weight	Risk Difference M-H, Fixed, 95% CI
Légaré 2012	53	163	43	165	100.0%	0.06 [-0.03, 0.16]
**Total (95% CI)**		**163**		**165**	**100.0%**	**0.06 [-0.03, 0.16]**
Total events	53		43			

Heterogeneity: Not applicable
Test for overall effect: Z = 1.29 (P = 0.20)

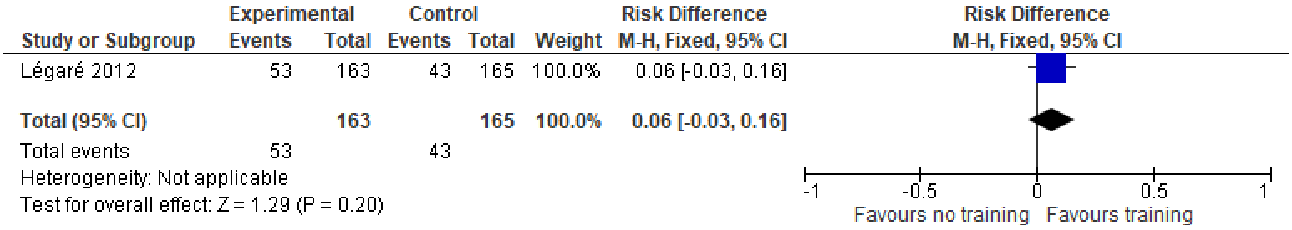

Figure A5-2: Shared decision making – patient reported outcome (categorical).

Healthcare professional reported outcome measure

Study or Subgroup	Experimental Mean	Experimental SD	Experimental Total	Control Mean	Control SD	Control Total	Weight	Mean Difference IV, Fixed, 95% CI
Légaré 2012	79.7	1.8	151	76.3	1.9	99	100.0%	3.40 [2.93, 3.87]
**Total (95% CI)**			**151**			**99**	**100.0%**	**3.40 [2.93, 3.87]**

Heterogeneity: Not applicable
Test for overall effect: Z = 14.13 (P < 0.00001)

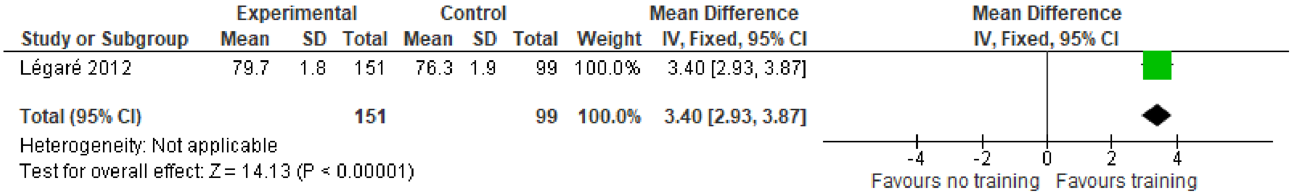

Figure A5-3: Shared decision making – healthcare professional reported outcome.

Decisional regret

Study or Subgroup	Experimental Mean	Experimental SD	Experimental Total	Control Mean	Control SD	Control Total	Weight	Mean Difference IV, Fixed, 95% CI
Légaré 2012	12.4	19.1	163	7.6	13.7	165	100.0%	4.80 [1.20, 8.40]
**Total (95% CI)**			**163**			**165**	**100.0%**	**4.80 [1.20, 8.40]**

Heterogeneity: Not applicable
Test for overall effect: Z = 2.61 (P = 0.009)

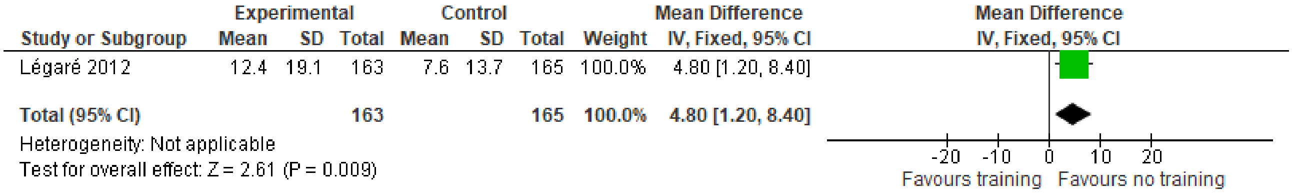

Figure A5-4: Decisional regret

Decisional conflict

Study or Subgroup	Experimental Events	Experimental Total	Control Events	Control Total	Weight	Risk Difference M-H, Fixed, 95% CI
Légaré 2012	8	163	11	165	100.0%	-0.02 [-0.07, 0.03]
**Total (95% CI)**		**163**		**165**	**100.0%**	**-0.02 [-0.07, 0.03]**
Total events	8		11			

Heterogeneity: Not applicable
Test for overall effect: Z = 0.68 (P = 0.49)

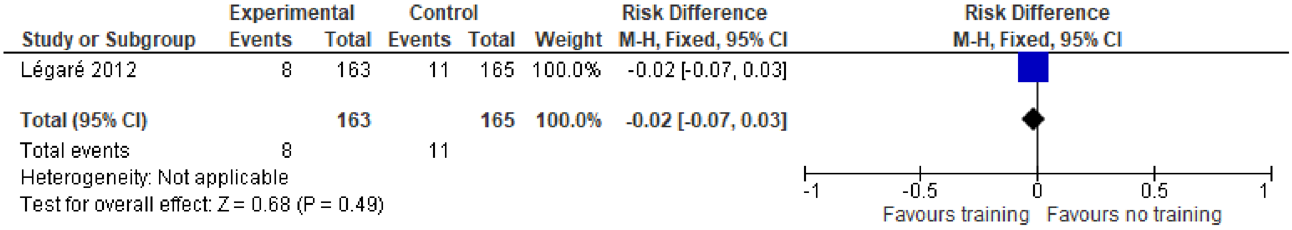

Figure A5-5: Decisional regret – patient view

Study or Subgroup	Experimental Events	Experimental Total	Control Events	Control Total	Weight	Risk Difference M-H, Fixed, 95% CI
Légaré 2012	8	160	2	108	100.0%	0.03 [-0.01, 0.07]
**Total (95% CI)**		**160**		**108**	**100.0%**	**0.03 [-0.01, 0.07]**
Total events	8		2			
Heterogeneity: Not applicable						
Test for overall effect: Z = 1.46 (P = 0.14)						

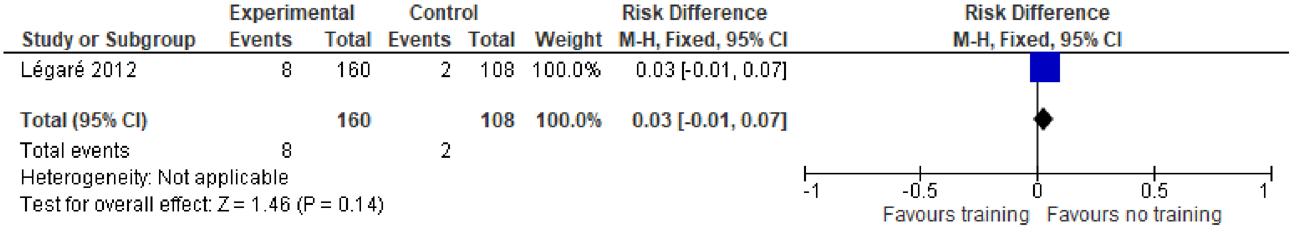

Figure A5-6: Decisional regret – physician view

Patients' intention to engage in SDM

Study or Subgroup	Experimental Mean	Experimental SD	Experimental Total	Control Mean	Control SD	Control Total	Weight	Mean Difference IV, Fixed, 95% CI
Légaré 2012	2.1	1.1	163	1.9	1.2	165	100.0%	0.20 [-0.05, 0.45]
**Total (95% CI)**			**163**			**165**	**100.0%**	**0.20 [-0.05, 0.45]**
Heterogeneity: Not applicable								
Test for overall effect: Z = 1.57 (P = 0.12)								

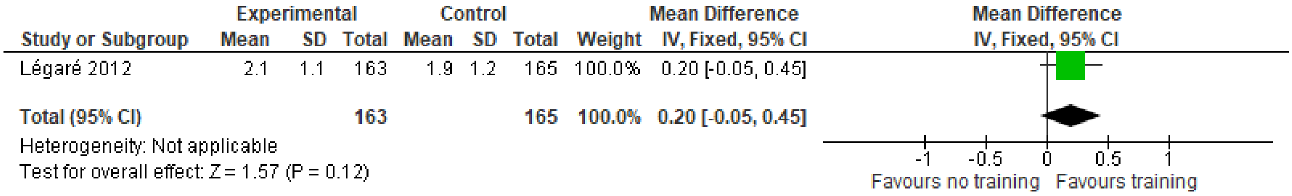

Figure A5-7: Patients' intention to engage in SDM

Quality of life

Study or Subgroup	Experimental Mean	Experimental SD	Experimental Total	Control Mean	Control SD	Control Total	Weight	Mean Difference IV, Fixed, 95% CI
**2.8.1 Physical**								
Légaré 2012	49.4	7.5	181	48.2	7.8	178	100.0%	1.20 [-0.38, 2.78]
**Subtotal (95% CI)**			**181**			**178**	**100.0%**	**1.20 [-0.38, 2.78]**
Heterogeneity: Not applicable								
Test for overall effect: Z = 1.49 (P = 0.14)								
**2.8.2 Mental**								
Légaré 2012	50.8	9.3	181	51.2	8.4	178	100.0%	-0.40 [-2.23, 1.43]
**Subtotal (95% CI)**			**181**			**178**	**100.0%**	**-0.40 [-2.23, 1.43]**
Heterogeneity: Not applicable								
Test for overall effect: Z = 0.43 (P = 0.67)								
Test for subgroup differences: $Chi^2 = 1.68$, df = 1 (P = 0.20), $I^2 = 40.4\%$								

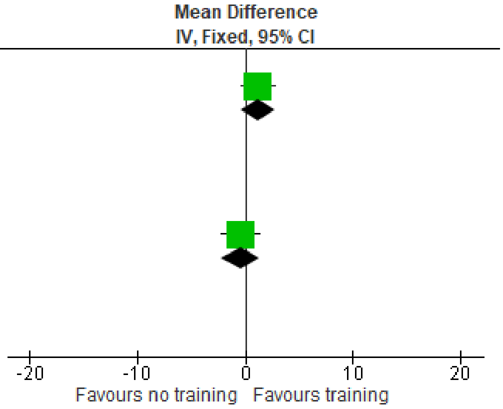

Figure A5-8: Quality of life.
