# Supplementary material for: A systematic review of shared decision making training programs for general practitioners
Source: BMC Med Educ. 2024 May 29;24:592. doi: 10.1186/s12909-024-05557-1 (PMC11137915; doi:10.1186/s12909-024-05557-1)
Supplement: Supplementary file 6 — Supplementary Material 6. [file 12909_2024_5557_MOESM6_ESM.pdf]

Additional file 6: Risk of bias

For section 2 on managing bias in sampling or between groups, 16 studies (55%) had a low risk, 11 studies (38%) an unclear risk and 2 studies (7%) high risk of bias. For assessing managing bias in outcome measurements and blinding, 8 studies (27%) were rated at low risk of bias, 15 studies (52%) were rated as unclear risk of bias and 6 studies (21%) at high risk of bias. Section 4, managing bias in follow up, was rated at being at low risk of bias in 19 studies (65%), unclear risk in 8 studies (28%) and high risk in 2 studies (7%). For section 5, managing bias in other study aspects, 19 studies (66%) were rated as having a low risk of bias and 10 studies (34%) were rated as having an unclear risk of bias. Section 6, analytical rigour, was rated at a low risk of bias in 17 studies (59%) and unclear risk in 12 studies (41%). For assessing section 7, managing bias in reporting/ethical considerations, 6 studies were rated as having a low risk of bias (21%) and 23 studies as unclear risk of bias (79%).

	Clear aims	Baseline measurements	Lack of control group explanation	Appropriateness qualitative methodology	Appropriate study design	Sequence generation	Allocation concealement	Justification sample choice	Protection against selection bias	Comparability of groups	Sampling and recruitment	Blinding	Selection bias	Contamination	Secular changes	Blinded assessmet of outcome measures	Reliable primary outcome measures	Comparability of outcomes	Follow-up of subjects	Follow-up of episodes of care	Incomplete outcome data addressed	Detection bias	Information bias	Data collection appropriate	Attempts to mitigate effects of no control	Sufficient data points	Shaping of intervention effects specified	Analysis free from bias	Free of selective outcome reporting	limitations addressed	Conlusions clear	Free or other bias	Ethics issues addressed
	1A	1B	1C	1D	1E	2A	2B	2C	2D	2E	2F	3A	3B	3C	3D	3E	3F	3G	4A	4B	4C	5A	5B	5C	5D	6A	6B	6C	7A	7B	7C	7D	7E
1 Adarkwah 2016 (30)	2	-	-	-	-	2	2	-	-	-	-	1	-	-	-	1	1	-	2	2	2	1	-	-	-	-	-	2	2	1	2	1	2
2 Bakhit 2018 (45)	2	-	-	-	-	2	2	-	-	-	-	1	-	-	-	1	1	-	2	2	2	2	-	-	-	-	-	2	2	2	2	1	2
3 Branda 2013 (46)	2	-	-	-	-	1	2	-	-	-	-	2	-	-	-	1	2	-	1	1	0	2	-	-	-	-	-	1	0	2	2	2	2
4 Cals 2007 (31)	2	-	-	-	-	2	2	-	-	-	-	1	-	-	-	0	1	-	2	2	2	2	-	-	-	-	-	2	1	2	2	2	2
5 Cooper 2011 (47)	2	-	-	-	-	2	2	-	-	-	-	0	-	-	-	1	0	-	2	2	2	2	-	-	-	-	-	2	2	2	2	2	2
6 Cooper 2013 (48)	2	-	-	-	-	2	2	-	-	-	-	2	-	-	-	2	0	-	2	2	2	2	-	-	-	-	-	2	0	2	2	2	2
7 Den Ouden 2022 (33)	2	-	-	-	-	1	2	-	-	-	-	1	-	-	-	1	1	-	1	0	1	2	-	-	-	-	-	1	0	2	2	1	2
8 Elwyn 2004 (32,68,69)	2	-	-	-	-	2	2	-	-	-	-	2	-	-	-	2	1	-	2	1	1	2	-	-	-	-	-	2	1	2	2	1	2
9 Feng 2013 (49)	2	-	-	-	-	2	1	-	-	-	-	2	-	-	-	1	1	-	2	1	1	2	-	-	-	-	-	2	2	2	2	0	2
10 Ngu 2022 (39)	2	-	-	-	-	2	2	-	-	-	-	1	-	-	-	2	2	-	2	2	2	2	-	-	-	-	-	2	1	2	2	2	2
11 Haskard 2009 (34)	2	-	-	-	-	2	1	-	-	-	-	1	-	-	-	1	1	-	2	2	2	1	-	-	-	-	-	1	1	2	2	1	2
12 Helitzer 2011 (35)	2	-	-	-	-	2	1	-	-	-	-	1	-	-	-	1	2	-	2	2	2	2	-	-	-	-	-	1	1	0	2	2	2
13 Krones 2008 (36,70)	2	-	-	-	-	2	1	-	-	-	-	1	-	-	-	1	0	-	0	0	1	2	-	-	-	-	-	1	1	2	2	1	2
14 Kunneman 2020 (50)	2	-	-	-	-	2	2	-	-	-	-	0	-	-	-	0	2	-	2	2	2	2	-	-	-	-	-	2	0	2	2	1	2
15 Kunneman 2022 (28)	2	-	-	-	-	2	2	-	-	-	-	1	-	-	-	2	2	-	2	1	1	1	-	-	-	-	-	1	2	2	2	2	2
16 Legare 2013 (56,57,71)	2	-	-	-	-	2	2	-	-	-	-	0	-	-	-	0	0	-	2	1	1	1	-	-	-	-	-	2	2	2	2	0	2
17 Loh 2007 (37)	2	-	-	-	-	2	1	-	-	-	-	1	-	-	-	1	0	-	1	2	1	1	-	-	-	-	-	1	1	2	2	1	2
18 Mathers 2012 (53)	2	-	-	-	-	2	2	-	-	-	-	0	-	-	-	0	1	-	2	2	2	2	-	-	-	-	-	2	1	2	2	2	2
19 Moral 2001 (38)	2	-	-	-	-	2	1	-	-	-	-	2	-	-	-	2	1	-	2	2	2	2	-	-	-	-	-	1	1	2	2	1	1
20 Price Haywood 2004 (58)	2	-	-	-	-	2	1	-	-	-	-	0	-	-	-	0	0	-	2	2	1	2	-	-	-	-	-	2	2	2	2	0	2
21 Roter 2012 (54)	2	-	-	-	-	0	0	-	-	-	-	1	-	-	-	0	0	-	1	1	1	1	-	-	-	-	-	2	0	2	2	1	2
22 Sanders 2017 (29)	2	-	-	-	-	2	1	-	-	-	-	1	-	-	-	2	0	-	0	0	1	2	-	-	-	-	-	2	1	2	2	1	2
23 Sepucha 2022 (51)	2	-	-	-	-	2	1	-	-	-	-	2	-	-	-	2	0	-	1	0	2	1	-	-	-	-	-	2	1	2	1	2	2
24 Stewart 2007 (40)	2	-	-	-	-	2	2	-	-	-	-	1	-	-	-	2	1	-	2	1	1	1	-	-	-	-	-	2	1	2	2	1	2
25 Tai Seale 2016 (41)	2	-	-	-	-	0	0	-	-	-	-	1	-	-	-	2	0	-	1	1	0	1	-	-	-	-	-	1	1	2	2	1	2
26 Tilburgs 2020 (42)	2	-	-	-	-	2	2	-	-	-	-	1	-	-	-	2	2	-	2	2	2	1	-	-	-	-	-	1	1	2	2	1	2
27 Tinsel 2013 (43)	2	-	-	-	-	2	2	-	-	-	-	2	-	-	-	2	1	-	2	1	2	2	-	-	-	-	-	2	1	2	2	0	2
28 Wilkes 2013 (52)	2	-	-	-	-	2	2	-	-	-	-	2	-	-	-	2	0	-	2	2	1	2	-	-	-	-	-	2	1	2	2	0	2
29 Wollny 2021 (44)	2	-	-	-	-	2	2	-	-	-	-	2	-	-	-	2	2	-	2	1	2	1	-	-	-	-	-	1	1	2	2	2	2

Figure A7: Risk of bias using ICROMS. 0 = high risk of bias; 1 = unclear risk of bias; 2 = low risk of bias. Risk of bias was assessed in duplicate and independently. For detailed information regarding ICROMS criteria see original publication (Zingg et al. 2016 (25)).
